# Genome-wide comparative analysis of the *Brassica rapa *gene space reveals genome shrinkage and differential loss of duplicated genes after whole genome triplication

**DOI:** 10.1186/gb-2009-10-10-r111

**Published:** 2009-10-12

**Authors:** Jeong-Hwan Mun, Soo-Jin Kwon, Tae-Jin Yang, Young-Joo Seol, Mina Jin, Jin-A Kim, Myung-Ho Lim, Jung Sun Kim, Seunghoon Baek, Beom-Soon Choi, Hee-Ju Yu, Dae-Soo Kim, Namshin Kim, Ki-Byung Lim, Soo-In Lee, Jang-Ho Hahn, Yong Pyo Lim, Ian Bancroft, Beom-Seok Park

**Affiliations:** 1 Department of Agricultural Biotechnology, National Academy of Agricultural Science, Rural Development Administration, 150 Suin-ro, Gwonseon-gu, Suwon 441-707, Korea; 2 Department of Plant Science College of Agriculture and Life Sciences, Seoul National University, San 56-1, Sillim-dong, Gwanak-gu, Seoul 151-921, Korea; 3 National Instrumentation Center for Environmental Management, College of Agriculture and Life Sciences, Seoul National University, San 56-1, Sillim-dong, Gwanak-gu, Seoul 151-921, Korea; 4 Vegetable Research Division, National Institute of Horticultural and Herbal Science, Rural Development Administration, Tap-dong 540-41, Gwonseon-gu, Suwon 441-440, Korea; 5 Korea Research Institute of Bioscience and Biotechnology, 111 Gwahangno, Yuseong-gu, Daejeon 305-806, Korea; 6 School of Applied Biosciences, College of Agriculture and Life Sciences, Kyungpook National University, Daegu 702-701, Korea; 7 Department of Horticulture, Chungnam National University, 220 Kung-dong, Yusong-gu, Daejon 305-764, Korea; 8 John Innes Centre, Norwich Research Centre, Colney, Norwich NR4 7UH, UK

## Abstract

**Background:**

*Brassica rapa *is one of the most economically important vegetable crops worldwide. Owing to its agronomic importance and phylogenetic position, *B. rapa *provides a crucial reference to understand polyploidy-related crop genome evolution. The high degree of sequence identity and remarkably conserved genome structure between *Arabidopsis *and *Brassica *genomes enables comparative tiling sequencing using *Arabidopsis *sequences as references to select the counterpart regions in *B. rapa*, which is a strong challenge of structural and comparative crop genomics.

**Results:**

We assembled 65.8 megabase-pairs of non-redundant euchromatic sequence of *B. rapa *and compared this sequence to the *Arabidopsis *genome to investigate chromosomal relationships, macrosynteny blocks, and microsynteny within blocks. The triplicated *B. rapa *genome contains only approximately twice the number of genes as in *Arabidopsis *because of genome shrinkage. Genome comparisons suggest that *B. rapa *has a distinct organization of ancestral genome blocks as a result of recent whole genome triplication followed by a unique diploidization process. A lack of the most recent whole genome duplication (3R) event in the *B. rapa *genome, atypical of other *Brassica *genomes, may account for the emergence of *B. rapa *from the *Brassica *progenitor around 8 million years ago.

**Conclusions:**

This work demonstrates the potential of using comparative tiling sequencing for genome analysis of crop species. Based on a comparative analysis of the *B. rapa *sequences and the *Arabidopsis *genome, it appears that polyploidy and chromosomal diploidization are ongoing processes that collectively stabilize the *B. rapa *genome and facilitate its evolution.

## Abstract 2

Euchromatic regions of the Brassica rapa genome were sequenced and mapped onto the corresponding regions in the Arabidopsis thaliana genome.

## Background

Flowering plants (angiosperms) have evolved in genome size since their sudden appearance in the fossil records of the late Jurassic/early Cretaceous period [1-4]. The genome expansion seen in angiosperms is mainly attributable to occasional polyploidy. Estimation of polyploidy levels in angiosperms indicates that the genomes of most (>90%) extant angiosperms, including many crops and all the plant model species sequenced thus far, have experienced one or more episodes of genome doubling at some point in their evolutionary history [5,6]. The accumulation of transposable elements (TEs) has been another prevalent factor in plant genome expansion. Recent studies on maize, rice, legumes, and cotton have demonstrated that the genome sizes of these crop species have increased significantly due to the accumulation and/or retention of TEs (mainly long terminal repeat retrotransposons (LTRs)) over the past few million years; the percentage of the genome made up of transposons is estimated to be between 35% and 52% based on sequenced genomes [7-12]. However, genome expansion is not a one-way process in plant genome evolution. Functional diversification or stochastic deletion of redundant genes by accumulation of mutations in polyploid genomes and removal of LTRs via illegitimate or intra-strand recombination can result in downsizing of the genome [13-15]. Nevertheless, neither of the aforementioned mechanisms has been demonstrated to occur frequently enough to balance genome size growth, and plant genomes tend, therefore, to expand over time.

The progress in whole genome sequencing of model genomes presents an important challenge in plant genomics: to apply the knowledge gained from the study of model genomes to biological and agronomical questions of importance in crop species. Comparative structural genomics is a well-established strategy in applied agriculture in several plant families. However, comparative analyses of modern angiosperm genomes, which have experienced multiple rounds of polyploidy followed by differential loss of redundant sequences, genome recombination, or invasion of LTRs, are characterized by interrupted synteny with only partial gene orthology even between closely related species, such as cereals [16], legumes [17,18], and *Brassica *species [19,20]. Furthermore, functional divergence of duplicated genes limits interpretation of function based on orthology, which complicates knowledge transfer from model to crop plants. Thus, better delimitation of comparative genome arrangements reflecting evolutionary history will allow information obtained from fully sequenced model genomes to be used to target syntenic regions of interest and to infer parallel or convergent evolution of homologs important to biological and agronomical questions in closely related crop genomes.

The mustard family (Brassicaceae or Cruciferae), the fifth largest monophyletic angiosperm family, consists of 338 genera and approximately 3,700 species in 25 tribes [21], and is fundamentally important to agriculture and the environment, accounting for approximately 10% of the world's vegetable crop produce and serving as a major source of edible oil and biofuel [22]. Brassicaceae includes two important model systems: *Arabidopsis thaliana *(*At*), the most scientifically important plant model system for which complete genome sequence information is available, and the closely related, agriculturally important *Brassica *complex - *B. rapa *(*Br*, A genome), *B. nigra *(*Bn*, B genome), *B. oleracea *(*Bo*, C genome), and their three allopolyploids, *B. napus *(*Bna*, AC genome), *B. juncea *(*Bj*, AB genome), and *B. carinata *(*Bc*, BC genome). Syntenic relationships and polyploidy history in these two model systems have been investigated, although details about macro- and microsyntenic relationships between *At *and *Brassica *are limited and fragmented. Previous studies demonstrated broad-range chromosome correspondence between the *At *and *Brassica *genomes [23,24], and a few studies have demonstrated specific cases of conservation of gene content and order with frequent disruption by interspersed gene loss and genome recombination [19,20]. Although this issue is contentious, there is evidence that Brassicaceae genomes have undergone three rounds of whole genome duplication (WGD; hereafter referred to as 1R, 2R, and 3R, which are equivalent to the γ, β, and α duplication events) [5,25,26]. One profound finding from comparative analyses is the triplicate nature of the *Brassica *genome, indicating the occurrence of a whole genome triplication event (WGT, 4R) soon after divergence from the *At *lineage approximately 17 to 20 million years ago (MYA) [19,20,26]. This result strongly suggests that comparative genomic analyses using single gene-specific amplicons or those based on small scale synteny comparisons will fail to identify all related genome segments, and thus not be able to provide accurate indications of orthology between the *At *and *Brassica *genomes. However, obtaining sufficient sequence information from *Brassica *genomes to identify genome-wide orthologous relationships between the *At *and *Brassica *genomes is a major challenge.

*Br *was recently chosen as a model species representing the *Brassica *'A' genome for genome sequencing [27,28]. This species was selected because it has already proved a useful model for studying polyploidy and because it has a relatively small (approximately 529 megabase-pair (Mbp)) but compact genome with genes concentrated in euchromatic spaces. However, widespread repetitive sequences in the *Br *genome hinder direct application of whole genome shotgun sequencing. Instead, targeted sequencing of specific regions of the *Br *genome could be informed by the reference *At *genome by selecting genomic clones based on sequence similarity; this approach is referred to as comparative tiling [29]. Here, we report sequencing of large-scale regions of the *Br *euchromatic genome, covering almost all of the *At *euchromatic regions, obtained using the comparative tiling method. We performed a genome-wide sequence comparison of *Br *and *At *and analyzed the number of substitutions per synonymous site (Ks) between the two genomes and among related *Brassica *sequences to identify syntenic relationships and to further refine our understanding of the evolution of polyploidy. We also investigated genome microstructure conservation between the two genomes. In this study, we provide a foundation to reconstruct both the ancestral genome of the *Brassica *progenitor and the evolutionary history of the *Brassica *lineage, which we anticipate will provide a robust model for *Brassica *genomic studies and facilitate the investigation of the genome evolution of domesticated crop species.

## Results

### Generation of *Br *euchromatic sequence contigs and genome coverage

**Figure 1:**
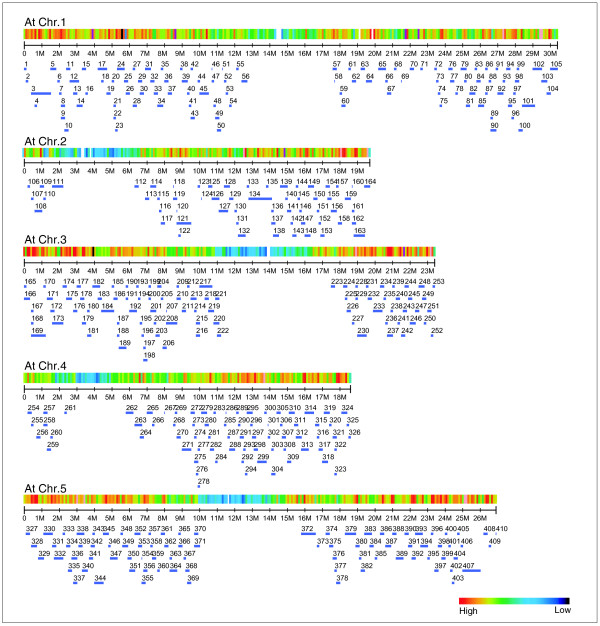
*In silico *allocation of 410 *B. rapa *BAC sequence contigs to *A. thaliana *chromosomes. BAC sequence contigs (blue bars) were aligned to *At *chromosomes based on significant and directional matches of sequences using a BLASTZ cutoff of <E^-6^.

Bacterial artificial chromosome (BAC) sequence assembly generated 410 *Br *sequence contigs (sequences composed of more than one BAC sequence) covering 65.8 Mbp (Tables S1 and S2 in Additional data file 1). These sequence contigs span 75.3 Mbp of the *At *genome, representing 92.2% of the total *At *euchromatic region (Figure 1 and Table 1). A total of 43.9 Mbp remain as uncovered gaps: among these, 6.4 Mbp are attributable to euchromatin gaps, and the remaining 37.5 Mbp to pericentromeric heterochromatin gaps.

**Table 1:** Summary of *B. rapa *chromosome sequences comparatively tiled on the *A. thaliana *genome

	*B. rapa*					
	
*A*. *thaliana*	Number of BACs	Number of sequence contigs	Total sequence length (Mbp)	Coverage of *At *genome (Mbp)	Gaps of *At *genome (Mbp)	
					
					Euchromatin	Heterochromatin
At1	147	105	16.5	18.5	1.4	10.5
At2	98	59	10.3	12.4	1.4	6
At3	124	89	14.2	15.7	0.4	7.4
At4	97	73	11.3	11.4	0.9	6.2
At5	123	84	13.5	17.3	2.3	7.4

Total	589	410	65.8	75.3	6.4	37.5

Sequence length and coverage were calculated according to Tables S1 and S2 in Additional data file 1.

The genome coverage of the gene-rich *Br *sequences was estimated by representation in two different datasets: expressed sequence tag (EST) sequences and conserved single-copy genes. Based on a BLAT analysis of 32,395 *Br *unigenes (a set of ESTs that appear to arise from the same transcription locus) against the sequence contigs, the proportion of hits recovered under stringent conditions (see Materials and methods) was 29.2%. This result was largely consistent with the proportion of rosid-conserved single-copy genes showing matches to *Br *sequences. A TBLASTN comparison of 1,070 *At*-*Medicago truncatula *(*Mt*) conserved single-copy genes against *Br *sequences revealed a 24.3% match. Both methods indicate approximately 30% coverage of euchromatin in the dataset analyzed; thus, the euchromatic region of *Br *is estimated to be approximately 220 Mbp, 42% of the whole genome given that the genome size of *Br *is 529 Mbp [30].

### Characteristics of the *B. rapa *gene space

Gene annotation was carried out using our specialized *Br *annotation pipeline. Gene prediction of the *Br *sequence data using a variety of *ab initio*, similarity-based, and EST/full-length cDNA-based methods resulted in the construction of 15,762 gene models. Taken together with the genome coverage of *Br *sequences, the overall number of protein-coding genes in the *Br *genome is at least 52,000 to 53,000, which is higher than those of other plant genomes sequenced thus far, including *At *[7], rice (*Oryza sativa *(*Os*)) [8], poplar (*Populus trichocarpa *(*Pt*)) [9], grape [10], papaya [11], and sorghum [12]. However, the estimated total number of genes in the *Br *genome is only twice that of *At*. Details of the annotation are available online at the URL cited in the 'Data used in this study' section in the Materials and methods.

The gene structure and density statistics are shown in Table 2. The base composition of *Br *and *At *genes is very similar. The average length of *Br *genes (ATG to stop codon) is 73% that of *At *genes. This is consistent with previous reports on *Bo *[19,20,26]. This difference appears to be due to one less exon per gene and shorter exon and intron lengths in *Br*. The average gene density of 1 per 4.2 kilobase-pairs (kbp) in *Br *is slightly lower than that in *At *(1 per 3.8 kbp). Thus, the *At*/*Br *ratio of gene density is 0.90, indicating slightly less compact organization of *Br *euchromatin than *At *euchromatin. Moreover, the distance between the homologous block endpoints in *Br *and *At *has an R^2 ^of 0.63 with a *dAt*/*dBr *slope of 1.36 (Figure S1 in Additional data file 2). This result indicates that gene-containing regions in *At *occupy approximately 30 to 40% more space than their *Br *counterparts. Based on these data and the results mentioned above, we postulate that the euchromatic genome of *Br *has shrunken by approximately 30% compared to its syntenic *At *counterpart. Most of the genome shrinkage in *Br *could be explained by the deletion of roughly one-third of the redundant proteome as well as TEs in the euchromatic *Br *genome. Only 14% of the *Br *genes were tandem duplicates compared with 27% of *At *genes in a 100-kbp window interval. In addition, only 45 nucleotide binding site-encoding genes were identified in *Br*, suggesting that the total number of nucleotide binding site-encoding genes in the *Br *genome is likely to be almost the same as that in *At *(approximately 200) [31,32]. A database search revealed that a total of 12,802 (81%) of the predicted *Br *genes have similarity (<E^-10^) to proteins in the non-redundant nucleotide database of the National Center for Biotechnology Information (NCBI); 2,960 (19%) are *Br *unique genes. To assess the putative function of the genes that recorded no hits to non-redundant proteins, we assigned functional categories to the *Br *unique genes using gene ontology analysis; however, this analysis could not identify a putative function for approximately 85% of the *Br *unique genes. Thus, we can conclude that 16% of the proteome of *Br *has acquired a novel function since the *Br*-*At *divergence.

**Table 2:** Comparison of the overall composition of annotated protein coding genes in the *B. rapa *sequence contigs and euchromatic counterparts in the *A. thaliana *genome

Feature	*B. rapa*	*A*. thaliana*
Number of sequence contigs	410	
Total sequence length (Mbp)	65.8	75.3
Transposons (%)	6	3
Number of protein coding genes	15,762	19,639
Number of exons per gene	4.7	5.5
Intron size (bp)	141	162
Exon size (bp)	225	230
Average gene size (kbp)	1.6	2.2
Average gene density (kbp/gene)	4.2	3.8
Overall G/C content (%)	35.2	35.8
Exons	46.3	44.6
Introns	32.6	32.0
Intergenic regions	31.3	31.8

*A. thaliana statistics are based on version TAIR7 annotation available on the *Arabidopsis *Information Resource website [74].

Repetitive sequence analysis revealed that 6% of euchromatic *Br *sequences are composed of TEs, a twofold greater amount than identified in the counterpart *At *euchromatic genome, presumably due to a greater number of LTRs and long interspersed elements (Table 3). In addition, low complexity repetitive sequences are relatively abundant in the *Br *euchromatic region, indicating *Br*-specific expansion of repetitive sequences. The distribution of repetitive sequences and TEs along the chromosomes was not uneven (Figure S2 in Additional data file 2). It has previously been reported, based on partial draft genome shotgun sequences, that *Bo *(approximately 696 Mbp) has a significantly higher proportion of both class I and class II TEs sequences than *At *[33]. Taken together with these previous reports [34,35], TEs appear to be partly responsible for genome expansion in the *Brassica *lineage, and these TEs appear to accumulate predominantly in the heterochromatic regions of *Br*.

**Table 3:** Comparison of repetitive sequences identified in the *B. rapa *sequence contigs and euchromatic counterparts in the *A. thaliana *genome

	Genome coverage (%)*	
	
Family	*B. rapa*	*A. thaliana*
SINEs	0.1	0.0
LINEs	1.3	0.3
LTRs	2.4	0.8
DNA transposons	2.2	1.6
Satellites	0.4	0.0
Low complexity repetitive sequences	4.4	1.0
Other^†^	0.4	0.1

Total	11.2	3.8

*Genome coverage was calculated using 65.8 Mbp for *B. rapa *and 75.3 Mbp for the euchromatic counterpart of *A. thaliana*. ^†^This refers to simple sequence repeats and short tandem repeats. LINE, long interspersed element; SINE, short interspersed element.

### Synteny between the *B. rapa *and *A. thaliana *genomes

**Figure 2:**
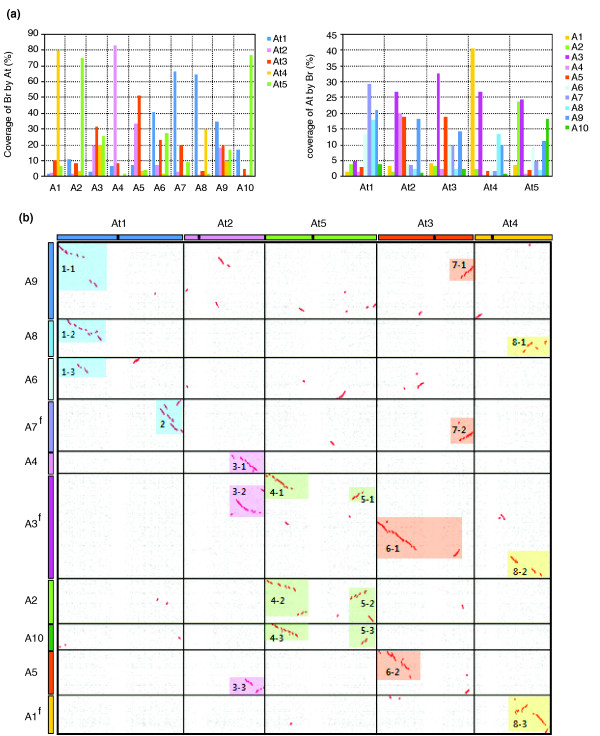
Synteny between the *B. rapa *and *A. thaliana *genomes. **(a) **Percent coverage of individual chromosomes showing synteny between *B. rapa *and *A. thaliana*. Coverage was calculated as the gene number of an individual chromosome per sum of genes with BLASTP hits. Note that the overall coverage of an individual chromosome for the counterpart genome can exceed 100% because multiple best BLAST hits over the same region are counted. **(b) **Chromosome correspondence between *B. rapa *and *A. thaliana *represented by a dot-plot. Each dot represents a reciprocal best BLASTP match between gene pairs at an E-value cutoff of <E^-20^. Red dots show regions of synteny with more than 50% gene conservation as identified by DiagHunter. Some *Br *chromosome orientations have been flipped (A1^f^, A3^f^, A7^f^) to visually correspond to *At *orientations. Both *Br *and *At *have been scaled to occupy the same lengths. Color bars on the upper and left margins of the dot plot indicate individual chromosomes of *At *and *Br*, respectively. Black dots on the *At *chromosomes are centromeres. The color-shaded boxes in the dot plots represent long-range synteny blocks along chromosome pairs. Boxes with the same color are putative triplicated remnants. See Additional data file 3 and the URL cited in Materials and methods for all dot plots and related results, including detailed close-ups of regions of synteny.

To identify syntenic regions in the *Br *and *At *genomes, we compared the whole proteome between the two genomes using BLASTP analysis, and putative synteny blocks were plotted using DiagHunter and GenoPix2D programs [36]. The non-redundant chromosome-ordered genome sequence in the *Br *build was 62.5 Mbp. An additional 3.2 Mbp had not yet been assigned to chromosomes and was therefore not used for synteny analysis. We examined the synteny blocks at three different levels: whole genome (Figure 2a), large-scale synteny blocks in chromosome-to-chromosome windows (Figure 2b; Additional data file 3), and microsynteny <2.5 Mbp (the synteny can be viewed at the URL cited in the 'Data used in this study' section in the Materials and methods). Although the *Br *genome build was partial and incomplete with only approximately 30% of euchromatin represented and some misordered contigs present, the level of synteny between the genomes was prominent and distinct. The DiagHunter program detected 227 highly homologous syntenic blocks with 72% of the sequenced and anchored *Br *sequence assigned to synteny blocks in *At *and 72% of *At *euchromatic sequence assigned to synteny blocks in *Br *when multiple blocks overlapping the same region were counted (Figure 2a). Considering the history of frequent genome duplication events in Brassicaceae, this result strongly indicates the presence of secondary or tertiary blocks resulting from WGT.

The *Br *and *At *genomes share a minimum of 20 large-scale synteny blocks with substantial microsynteny; these synteny blocks extend the length of whole chromosome arms. *At *shows synteny of chromosome arms with multiple chromosome blocks of *Br*, apparently corresponding to triplicated remnants (Figure 2b). *At*1S (short arm), *At*2L (long arm), *At*4L, and *At*5 have three long-range synteny counterparts in three independent *Br *chromosomes. However, *At*1L and *At*3 have only one or two synteny blocks in the *Br *genome. Moreover, some genome regions of *At*, including a smaller section of *At*2S and *At*4S, show no significant synteny with *Br *counterparts, indicating chromosome-level deletion of triplicated segments. Incidentally, *Br *shows synteny with a major single chromosome along almost the entire length (A1, A2, A4, and A10) or fragments of multiple *At *chromosomes in a complicated mosaic pattern, indicating frequent recombination of *Br *chromosomes. Notable regions of synteny are shown in Figure 2b, and are At1S-A6/A8/A9, At1L-A7, At2L-A3/A4/A5, At3S-A3/A5, At3L-A7/A9, At4L-A1/A3/A8, and At5-A2/A3/A10 (synteny view available at the URL cited in the 'Data used in this study' section in the Materials and methods. Additional synteny blocks scattered throughout genome regions, probably due to recombination, were also identified.

Within individual synteny blocks, microsynteny (conservation of gene content and order) was considerable. The average degree of proteome conservation for all predicted synteny blocks was 52 ± 13% in the blocks (Table S3 in Additional data file 1). This value is almost the same as that of the *Mt*-*Lotus japonicus *comparison in which an ancient WGD event at a similar time period (Ks 0.7 to 0.9) as the *Br*-*At *WGD but earlier speciation (Ks 0.6) than *Br*-*At *was detected [18]. The underestimated value reported here presumably reflects significant gene loss and rearrangement after WGT in the *Br *lineage resulting in genome shrinkage, based on the fact that deletion events in syntenic blocks of the *Br *genome were twofold more frequent than in the *At *genome. Genes without corresponding homologs in syntenic regions contributed to 15 ± 7% of all genes from *Br *but 33 ± 13% from *At *(Table S3 in Additional data file 1; Additional data file 3). Genes encoding proteins involved in transcription or signal transduction were not found to be significantly more retained in syntenic blocks than those encoding proteins classified as having other functions. Further genome sequencing will help resolve the synteny in the uncovered and/or the scattered genome regions.

### Rearrangement of the *B. rapa *genome

**Figure 3:**
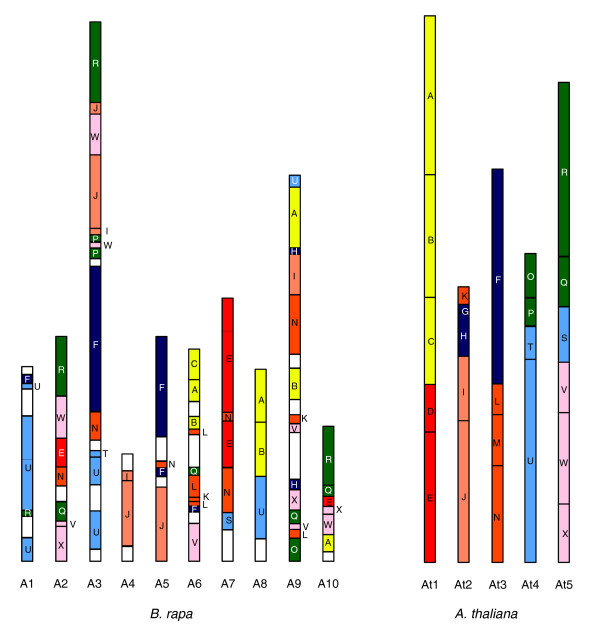
Comparison of the genome structures of *B. rapa *and *A. thaliana *based on 24 ancestral karyotype genome building blocks. The genome structure of *At *was based on the reports of Schranz *et al*. [37] and Lysak *et al*. [38]. The position of genome blocks in the *Br *chromosome was defined by a comparison of *Br*-*At *syntenic relationships and the *At*-*AK *mapping results. *Br *sequences were connected to form continuous sequences. Block boundaries, orientation, and gaps between syntenic blocks are shown in Additional data file 4. Each color corresponds to a syntenic region between genomes. The *Br *genome is triplicated and more thoroughly rearranged than the *At *genome.

Comparison of the genomes of *Br *and *At *allows insight into the origin and evolution of the *Brassica *'A' genome. Previous comparative mapping studies have identified a putative ancestral karyotype (*AK*) comprising 24 building blocks on 8 chromosomes from which the current *Arabidopsis *and *Brassica *genomes have evolved via fusion/fission, rearrangement, and deletion of chromosomes followed by polyploidy [23,37-39]. According to the *At*-*AK *relationship and pair information of *Br*-*At *synteny blocks, we defined conserved genome building blocks of *AK *on the *Br *genome build (Figure 3; Additional data file 4). The pattern of block boundaries on *Br *chromosomes was similar to that reported pattern for *Bna *'A' genome components, albeit more complicated (Figure S3 in Additional data file 2). Most of the block boundaries were conserved between *Br *and the 'A' genome components of *Bna *with the exception of several insertions/deletions; this is presumably due to limited sequence and marker information. In addition, inversion or serial mismatched block boundaries were found on A2, A7 and A9, respectively, suggesting recombination of homologous counterpart regions between the 'A' and 'C' genomes in *Bna*.

An examination of the *Br *genome from the perspective of ancestral blocks reveals that three copies of the genome are present, as predicted from the WGT (Figure 3). Although there are several discontinuous matches due to gaps between syntenic blocks, almost 50% of the ancestral blocks were triplicated in the *Br *genome, while others occurred only once or twice, indicating loss of blocks during genome rearrangement. Blocks D, G, and M could not be found on the *Br *genome. The *Br *genome is highly rearranged relative to *At *compared with *AK*. Block R was localized together with block W in triplicate regions (A2, A3, and A10). However, in At5, blocks R and W were separated on the short arm and long arm, respectively [38,39]. Similarly, blocks E and N were adjacent and triplicated in *Br *but separated in *At*. Meanwhile, blocks K and L, which are fused in *AK *but split in different chromosomes of *At*, were adjacent (A6) or separated (A9) on the same chromosomes of *Br*. However, we did not determine precisely which copy of the replicated *AK *block family corresponds to the *Br *BACs because of the possibility that *Br *sequences in the polyploid genome were not accurately positioned. Because several genetic markers originate from duplicate or triplicate regions of the *Br *genome, the true location of the BACs could correspond to any of the amplified bands, which could result in inaccurate mapping of the BAC sequence. In this case, the resulting assignment of the BAC to an incorrect linkage group on a specific *AK *block family member would also be flawed; however, we found that almost all BAC sequences showed excellent correspondence to the correct family of *AK *blocks. Further analysis, including chromosome painting and additional genome sequencing, will allow determination of the precise location of *AK *blocks in the *Br *genome.

### Loss of genes from the recent duplication event in the *B. rapa *genome

**Figure 4:**
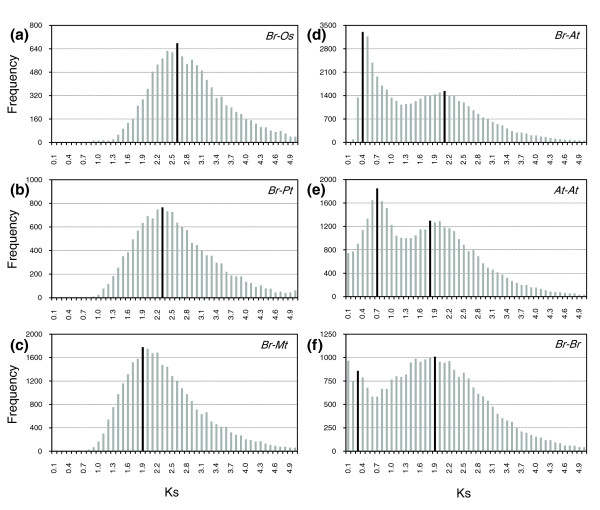
Traces of polyploidy events in plant genomes. **(a-f) **The distribution of Ks values obtained from comparisons of sets of putative orthologous genome sequences between *Br *and the selected model plant species *Os *(a), *Pt *(b), *Mt *(c), and *At *(d), and from paralogous sequences in *At *(e) and *Br *(f) genomes. The vertical axes indicate the frequency of paired sequences, while the horizontal axes denote Ks values with an interval of 0.1. The black bars depict the positions of the modes of Ks distributions obtained from orthologous or paralogous gene pairs. *At*, *A. thaliana*; *Br*, *B. rapa*; *Mt*, *Medicago truncatula*; *Os*, *O. sativa*; *Pt*, *Populus trichocarpa*.

To deduce the approximate time point of polyploidy and speciation, we compared the distribution of synonymous substitution (Ks) in homologous sequences identified by a reciprocal best BLAST hit search between *Br *and the completely annotated sequences of *At*, *Pt*, *Mt*, and *Os*. As shown in Figure 4a-c, *Br *shares a single ancient duplication event (1R) with *Os*, *Pt*, and *Mt *as illustrated by single peaks at Ks modes of 2.5 to 2.6, 2.2 to 2.3, and 1.8 to 1.9, respectively, indicating successive splitting of the *Br *lineage from monocots and eurosid I during the early and late Cretaceous period around 60 to 120 MYA, depending on the neutral substitution rate used [40]. The age distributions of *At *and *Br *yield clear peaks corresponding to 2R at Ks = 1.7 to 1.8 and 1.8 to 1.9, respectively, lower than that of the *Br*-*Pt *comparison but similar to that of the *Br*-*Mt *comparison (Figure 4e, f). This suggests that an ancient burst of gene duplications due to the 2R event in *At *and *Br *must have occurred almost immediately after divergence between eurosid I and eurosid II. Taken together with recent studies of the *Pt *[9] and *Mt *genomes [18], we conclude that genome duplication in rosids occurred independently after the split from the last common rosid ancestor, and that most polyploidy events (2R, 3R, and 4R) in Brassicaceae postdate the eurosid I (*Pt *and *Mt*)-eurosid II (*At *and *Br*) divergence.

The Ks distribution for *At *and *Br *orthologs displayed two peaks at Ks = 0.3 to 0.4 and 2.0 to 2.1, corresponding to shared duplication events (3R and 2R) and speciation between the genomes at around 13 to 17 MYA (Figure 4d). As reported before, the oldest duplication (1R) could not be seen in the Ks distributions in both genomes. Surprisingly, a comparison of the Ks mode for the paralogs in *At *and *Br *identified remarkable differences in the duplicated genes retained in the two genomes. Furthermore, the *At *genome has two clear peaks for 3R (mode Ks = 0.6 to 0.7) and 2R (mode Ks = 1.7 to 1.8). However, in the *Br *genome, two peaks representing 4R (mode Ks = 0.2 to 0.3) and 2R (mode Ks = 1.8 to 1.9) are evident, but the 3R peak has collapsed (Figure 4e, f). The difference between the distributions for *Br*-*Br *versus *Br*-*At *(*P *= 1.65E^-8^) was significantly higher than that for *At*-*At *versus *Br*-*At *(*P *= 0.001). Taken together, these findings suggest that duplicated genes produced by the 3R event were widely lost in the triplicated *Br *genome.

**Figure 5:**
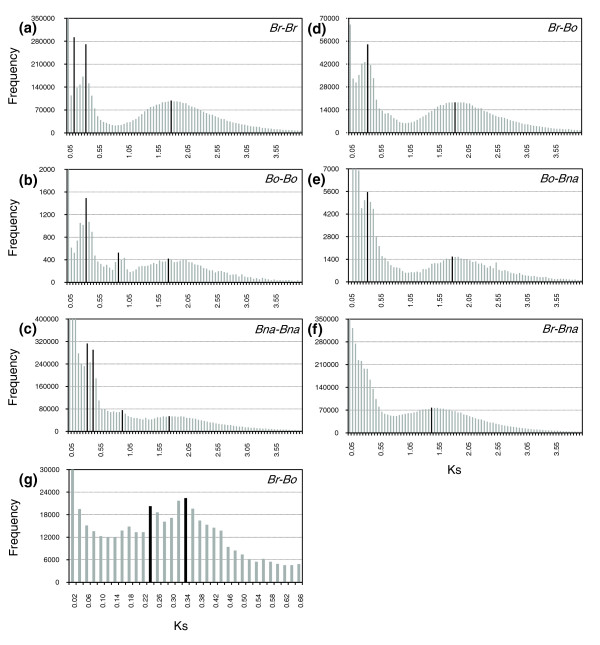
Traces of polyploidy events in the *Brassica *'A' and 'C' genomes. **(a-g) **Distributions of Ks values were obtained from comparisons of sets of paralogous EST sequences in *Br *(a), *Bo *(b), and *Bna *(c) and comparisons of putative orthologous EST sequences between the genomes (d-g). The vertical axes indicate the frequency of paired sequences, and the horizontal axes denote Ks values at 0.05 (a-f) or 0.02 (g) intervals. The black bars indicate the positions of the modes of Ks distributions obtained from comparisons of orthologous or paralogous gene pairs. *Bna*, *B*. *napus*; *Bo*, *B. oleracea*; *Br*, *B. rapa*.

Because we used approximately 30% of the euchromatic sequence of *Br*, we could have underestimated the 3R event due to biased sampling. To test this possibility, we analyzed the Ks distribution using ESTs. The age distribution of *Br *based on approximately 120,000 ESTs showed a pattern essentially identical to that obtained using the genome sequence data, illustrating loss of the 3R peak (Figure 5a). The additional peak for Ks = 0.10 to 0.15 may represent a very recent segmental duplication event. Loss of the 3R event appears to be specific to *Br *amongst *Brassica *genomes (Figure 5b-f); a *Bo*-*Bo *comparison yielded a Ks distribution different to that of *Br*-*Br*, with a clear peak corresponding to 3R (mode Ks = 0.85 to 0.90). A similar pattern was observed in the *Bna*-*Bna *comparison with underestimation of the peaks for 3R. However, note that the Ks modes for ortholog comparison between *Br *and *Bo*, *Bo *and *Bna*, and *Br *and *Bna *showed very similar Ks distribution with the two peaks for 4R and 2R at similar Ks modes as those in *Br*-*Br *paralog analyses, but loss of a peak for 3R. In particular, when the interval of Ks for the *Br*-*Bo *comparison was magnified, one additional peak, lying slightly below that for 4R at Ks = 0.34 to 0.36, was identified at Ks = 0.22 to 0.24; this indicates the genome split at around 8 MYA (Figure 5g).

Detection of a peak reflecting 3R in the *Bo *and *Bna *genomes but absence of this peak in the *Br *genome and between the other *Brassica *genomes strongly supports the hypothesis that duplicated genes from the 3R event were lost in the *Br *genome due to gradual deletion or suppression, presumably due to functional redundancy in the polyploid genome. To further explore this hypothesis, we compared the degree of conservation of duplicated genes in the sister blocks resulting from 3R and 4R. We found that 33 and 18 sister block pairs were selected for in the 3R and 4R events in the *Br *genome, respectively (Table S4 in Additional data file 1). The degree of conservation of duplicated genes for 4R was 44%, almost the same as that of the triplicated *FLC *region [20], but only 20% for 3R, a value approximately twofold lower than that of *Bo *based on calculations from published data [19]. This suggests greater deletion of duplicated genes in *Br *than *Bo *(Table 4; Tables S4 and S5 in Additional data file 1).

**Table 4:** Comparison of the degree of conservation between duplicated groups originating from different polyploidy events in *B. rapa *and *B. oleracea*

	Number of groups produced	Number of genes*			Degree of conservation
			
Duplication event		Total	Unpaired	Conserved	(%)^†^
*B. rapa*					
3R event	33	2,017	1,623	394	19.5
4R event	18 (3)	651 (112)	367 (58)	284 (54)	43.6 (48.2)
Segmental duplication	1 (1)	24 (28)	4 (0)	20 (28)	83.3 (100)
					
*B. oleracea*^‡^					
3R event	9	310	196	114	36.8
4R event	6	217	81	136	62.7

Data in parentheses are those from the *Flowering Locus C *(*FLC*) regions [20]. *Tandem duplicated genes were considered to be a single homolog. ^†^The degree of conservation was calculated by dividing the number of conserved genes by the total number of genes. ^‡^Information about the *Bo *genome was obtained from the report of Town *et al*. [19].

## Discussion

### A comparative genomics approach to target the euchromatic gene space of a crop genome

Investigation of crop genomes not only offers information that can be used for agricultural improvement, but also provides opportunities to understand angiosperm biology and evolution. As of 2009, the genome sequences of only five economically important crop plants (rice, poplar, grape, papaya, and sorghum) have been published [8-12], and whole genome sequencing projects are currently underway for only a few selected crop species. One hurdle faced when sequencing a crop genome is genome obesity due to polyploidy and repetitive DNA [41]. Therefore, a stepwise approach is required to obtain genome-wide information from crop genomes, and strategies for targeting gene-rich fractions are required. In combination with EST sequencing, two approaches - methylation filtration [42] and Cot-based cloning and sequencing [43] - were developed to capture euchromatic regions. Although both methods enrich for gene-rich fractions, they can exclude transcriptionally suppressed regions or euchromatic regions with abundant interspersed repetitive sequences (tandem repeats). We applied a novel gene space targeting method by allocating BAC clones to a closely related model genome based on BAC end sequence (BES) matches; this approach has not previously been reported in a genome sequencing project. This method has several advantages. First, gene-rich fractions of the crop genome can be obtained successfully *in silico *without additional experiments. We collected approximately 30% of the euchromatic region of *B. rapa *in this study. If a greater overlap between the clones and target region is allowed, and additional information in the form of genetic maps and physical contigs is used, the gene-rich fraction recovered is likely to increase significantly. Second, clone-by-clone strategies used in genome sequencing can benefit directly from this method because of selection of gene-rich seed BACs as well as the alignment of sequence scaffolds. Quick selection of a sufficient number of gene-rich seed BACs and directed ordering of the sequence scaffold will likely accelerate clone-based whole genome sequencing at reduced cost. The BAC clones selected in this study can be used as seed BACs for the ongoing clone-by-clone genome sequencing of *Br *[27,28]. Third, this analysis allows investigation of syntenic relationships between wild and crop genomes, thereby informing our understanding of crop evolution. Integration of genomes based on sequence level comparisons can offer a platform for the correlation between specific genes and phenotypes, which is important for further improvement of crops. We anticipate that application of our method will accelerate knowledge spreading from nodal model species to closely related taxa. For example, genome sequencing of other *Brassica *crops, particularly the construction of sequence assemblies and scaffolds of *Bna*, can benefit from the information obtained from the *Br *genome; this holds true even for next-generation sequencing. Thus, we anticipate that this study will make a significant contribution to structural and comparative genomic studies of crop species.

### Counterbalancing genome obesity after whole genome triplication in *B. rapa*

A large-scale comparison of *Br *genomic sequences and the whole euchromatic region of *At *demonstrated extensive synteny between the genomes, and provided clear evidence of a recent WGT event in the *Brassica *lineage. Our results significantly expand on previous observations of synteny between *At *and *Br *based on comparative genetic mapping [23] and small-scale comparisons of homologous regions [20] by deciphering the start-end points of macrosynteny blocks and elucidating the fine-scale details of microsynteny within the syntenic regions more accurately. Even though the *Br *sequencing project is still underway and the sequences used in this study are incomplete, the scale of synteny between the two genomes at both the macro- and micro-levels is significant. As the *Br *sequencing project moves forward, the availability of nearly complete coverage of the euchromatin will enable more precise definition of syntenic blocks between *At *and *Br*, which can be used to reconstruct ancestral chromosome sequences of *Brassica*.

Despite the WGT event, the total number of genes in the *Br *genome was estimated to be approximately 53,000, which is only a twofold increase compared with that of *At*. The usual fate of a duplicate-gene pair in a polyploid genome is nonfunctionalization or the deletion of one copy [44-46]. The reduction in the overall number of genes in the triplicated *Br *genome can be regarded as a result of a process that restores the diploid state, thereby counterbalancing genome obesity. This process seemed to be driven by the deletion of redundant genome components at the level of both the chromosome and the gene. A genome-wide synteny comparison between *Br *and *At *revealed that some of the triplicated copies of *Br *segments were lost or reconstructed. In addition, microsynteny analysis also indicated a relatively shrunken genome throughout the entire euchromatic region of the *Br *genome, with the *Br *gene space occupying a fraction 30% smaller than that of *At *due to a higher frequency of deletion events in the *Br *genome. A previous study reported that in the *At *genome, genes with regulatory functions, such as those encoding transcription factors or genes involved in signal transduction, were retained significantly more often than genes with other molecular functions [5]. However, we did not find differential retention of genes according to molecular function, which suggests random deletion of redundant genes in triplicated regions of the *Br *genome before functional diversification.

Several mechanisms responsible for post-polyploid changes have been proposed. These include chromosome rearrangements caused by unequal crossing-over, homologous recombination, translocation, or other cytogenetic events [47-50]. A tandem array with high sequence similarity would be a good candidate for deletion, because it is more likely to recombine and less likely to have a severe phenotype when one redundant gene is deleted. Fewer tandem duplicate genes in the *Br *genome may, therefore, be attributable to an increase in the rate of deletion. Incidentally, because polyploidy itself is a form of genomic 'disturbance,' it might induce a cellular response such as epigenetic silencing by hypermethylation, which may be especially relevant to genome evolution [48]. As a result, the epigenetic response itself may accelerate the rate of mutation, thereby causing rapid genomic change as seen in *Br*. In addition, polyploidy could increase transposable element activity, causing the deletion of genes or even chromosome segments. Illegitimate recombination of TEs has been demonstrated to have the ability to remove large blocks of DNA in *Arabidopsis*, rice, [15,49] and wheat [51]. We speculate that the twofold increase in transposon accumulation in the triplicated euchromatic regions of *Br *compared to the euchromatic counterpart regions of *At *might be correlated with the deletion of duplicated genes.

### Evolution of the *Brassica *'A' genome

**Figure 6:**
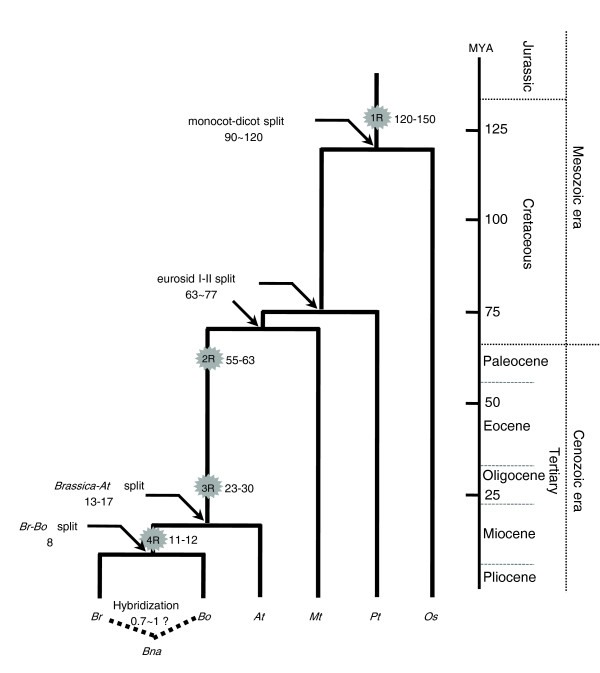
Polyploidy events in the evolution of the *Brassica *genome. Each star indicates a WGD (1R, 2R, and 3R) or WDT (4R) event on the branch. Estimation of dates for polyploidy and speciation events are given in million years and are based on the Ks analysis performed in this study, except for the 1R event, which was inferred from a previous report [1]. A geographic time-table is provided on the right border of the figure. *At*, *A. thaliana*; *Bna*, *B*. *napus*; *Bo*, *B. oleracea*; *Br*, *B. rapa*; *Mt*, *Medicago truncatula*; *Os*, *O. sativa*; *Pt*, *Populus trichocarpa*.

Multiple rounds of polyploidy are thought to have occurred during angiosperm evolution, although the number and timing of polyploidy events vary between plant groups [5,52]. Thus, most modern plant genomes harbor evidence of multiple rounds of past polyploidization. The genome evolution of Brassicaceae has been inferred mainly from studying *Arabidopsis*. There is evidence from several studies for one round of genome duplication after the eudicot divergence and additional rounds of polyploidization following the divergence of *Arabidopsis *from its common ancestor with cotton [5]. In this study, we refined inferences of the number and timing of polyploidy events, and we now discuss the impact of these events on the structure and evolution of the *Brassica *'A' genome (Figure 6). Ks estimates suggest that the *Brassica *genome shared two genome duplication events (2R and 3R) with *Arabidopsis *postdating the eurosid I (*Pt *and *Mt*) and eurosid II (*At *and *Br*) divergence. The third polyploidy event (4R) was a *Brassica *lineage-specific whole genome triplication after the split of *Brassica *from the common ancestor of *Brassica *and *Arabidopsis*. The 227 synteny blocks identified between *Br *and *At *can serve as a basis for reconstruction of the ancestral genome and chromosomes of the *Br*-*At *ancestor, although a more complete genome sequence and additional evidence are still required. The mapping of ancestral chromosome building blocks to the *Br *genome strongly suggests that the *Br *genome evolved from a pre-triplicated ancestor with a unique organization of the retracted *AK*, which was different from that of *At*, by chromosomal rearrangement shortly after 3R but prior to 4R. This event might have resulted in the divergence of the *Arabidopsis *and *Brassica *lineages.

More importantly, differential gene loss following 4R in the *Brassica *genome might be responsible for the diversification of the genome, based on the finding that significantly more genes duplicated as a result of 3R have been lost in *Br *than in *Bo*. However, it is not clear if duplicated genes from the 3R event that were retained in *Bo *have diverged functionally. It appears that the split between *Br *and *Bo *happened rapidly (0.1 Ks interval) compared to the *At*-*Brassica *split (0.3 Ks interval), perhaps due to differential retention of duplicated genes and genome recombination in the ancestral *Brassica *genome. These observations, along with the independent accumulation of repetitive sequences, may have facilitated speciation within the tribe *Brassiceae*, which contains approximately 240 highly diverse species. Further analysis and cross-comparisons of diploid and allopolyploid genomes of *Brassica *will enhance our understanding of the fate of duplicated genes in the *Brassica *genome. It appears that, as a counterbalance to genome obesity, there was higher selection pressure on redundant genes in the triplicated *Brassica *ancestor, accelerating gene loss in this triplicated ancestor compared to the *Arabidopsis*-*Brassica *common ancestor. Alternatively, differences in the life cycles of *Brassica *progenitors might have resulted in the differential deletion of duplicated genes in *Brassica *genomes. Moreover, artificial selection after domestication could also have had an impact on differentiation of diploid *Brassica *genomes. Taken together, the available evidence suggests that genome duplication and chromosomal diploidization are ongoing processes collectively driving the evolution of *Brassica *genomes.

## Conclusions

Comparisons of large-scale genomic sequences of *Br *and the whole euchromatic region of *At *revealed extensive synteny between the genomes due to at least two shared genome duplication events and a recent WGT event specific to the *Brassica *lineage. The reduction of the number of genes in the triplicated *Br *genome by approximately one-third can be regarded to be the result of a process counterbalancing genome obesity to regain the diploid state. Segmental loss of triplicated genome blocks and differential deletion of duplicated genes in *Br *along with less accumulation of transposons appear to have resulted in the small size of the *Br *genome (approximately 529 Mbp) compared to its sibling species, *Bo *(approximately 696 Mbp) and *Bn *(approximately 632 Mbp) [30]. The events proposed here indicate that genome diploidization following polyploidy played an important role in the radiation of *Brassica*. Our results clarify the orthology between *Br *and *At *and establish a strong basis for the genome evolution of *Brassica*. All the sequenced BAC clones investigated in this study were provided to the *B. rapa *Genome Sequencing Project as seed BACs for use as starting points for chromosome sequencing.

## Materials and methods

### BAC selection, sequencing, and sequence contig assembly

We previously published an efficient and novel clone selection method based on *in silico *BES matches to a model genome, which we named the comparative tiling method [29]. To select gene-rich *Br *BAC clones covering the entire *At *euchromatic regions, a total of 92,000 BESs were allocated to *At *chromosomes by using BLASTZ at a cutoff of <E^-6 ^with both end matches at 30 to 500 kbp intervals. A total of 4,647 BAC clones were allocated to 92 Mbp of *At *euchromatic regions and 589 minimally overlapping BAC clones (292 overlapping clones with an average of 41 kbp overlaps and 297 singleton clones) were finally selected and sequenced using an ABI 3730xl sequencer. The minimal sequence goal was five phase 2 (fully oriented and ordered sequence with some small gaps and low quality sequences) contigs, but 18 clones (3%) were sequenced as phase 1 due to large repetitive sequences (Table S1 in Additional data file 1). To anchor clones, a combination of sequence-based genetic mapping [53], fingerprint contig data [54], and fluorescent *in situ *hybridization (FISH) was used (Table S2 in Additional data file 1). The sequence contig assembly was created based on overlapping sequences. BAC sequences were assembled into big sequence contigs by first comparing paired BES matches and BAC sequences sharing overlapping positions on the target *At *chromosomes using Pipmaker [55]. Then, sequence contigs were assembled based on overlapping sequences using Phred/Phrap/Consed programs [56-58]. The location of sequence contigs or BAC singletons was determined primarily by genetic marker anchors with fingerprint contig information, paired BES, and FISH results providing additional information about local contig and BAC ordering. Pseudochromosome sequences were created by connecting sequence assemblies with 10-kbp additions of anonymous sequences. All the *Br *sequences used in this study are available at NCBI and the URL cited below in the 'Data used in this study' section and relevant reference sequence sources are listed in Table S6 in Additional data file 1.

### Estimation of genome coverage and genome annotation

The sequence coverage of the *Br *genome by BACs was estimated by calculating the proportions of *Br *EST unigenes and conserved single-copy rosid genes with strong matches. For EST comparisons, we considered unigenes to have a genome match if more than 90% of unigenes matched with at least 95% identity in a BLAT [59] analysis. For the single-copy rosid gene comparison, we created a list of 1,070 single-copy *At *and *Mt *genes not included in the *Br *EST collections. They were considered to have a genome match in *Br *if at least 50% of the gene matched in a TBLASTN [60] search at a cutoff of <E^-100^. The assembled sequences were masked using RepeatMasker [61] using a dataset combining the plant repeat element database of the Munich Information Center for Protein Sequence (MIPS) [62] and our specialized database of *Br *repetitive sequences. Gene model prediction was performed using EVidenceModeler [63]. Putative exons and open reading frames were predicted *ab initio *using FGENESH [64] and AUGUSTUS [65] programs with the parameters trained using the *Br *matrix. To predict consensus gene structures, *Br *ESTs plus full-length cDNAs, plant transcripts, and plant protein sequences were aligned to the predicted genes using PASA [66] and AAT [67] packages. The predicted genes and evidence sequences were then assembled according to the weight of each evidence type using EVidenceModeler. The highest scoring set of connected exons, introns, and noncoding regions was selected as a consensus gene model. Proteins encoded by gene models were searched against the Pfam database [68] and automatically assigned a putative name based on conserved domain hits or similarity with previously identified proteins. Annotated gene models were also searched against a database of plant transposon-encoded proteins [69]. Predicted proteins with a top match to transposon-encoded proteins were excluded from the annotation and gene counts.

### Identification of syntenic blocks based on genome comparisons

Syntenic regions of the genomes of *Br *and *At *were identified by a proteome comparison based on BLASTP [60] analysis. The entire proteomes of the two genomes were compared, and only the top reciprocal BLASTP matches per chromosome pair were selected (minimum of 50% alignment coverage at a cutoff of <E^-20^). We chose to perform a BLASTP similarity search because it is inherently more sensitive than BLASTN [60]. Moreover, the BLASTP hit matrix contains fewer BLAST hits that are due to repetitive nucleotide sequences. Chromosome scale synteny blocks were inferred by visual inspection of dot-plots using DiagHunter with parameters as described in Cannon *et al*. [36]. Gene orientation, insertions/deletions, and inversions were considered, and at least four genes with the same respective orientations in both genomes were required to establish a primary candidate synteny block. To distinguish highly homologous real synteny blocks from false positives due to multiple rounds of polyploidy followed by genome rearrangement, we manually checked all the primary candidate blocks. Previous studies reported that the degree of gene conservation between *At *and the *Brassica *genome in several selected syntenic regions was >50%. Based on this result, 227 blocks showing a gene conservation index of >50% (twice the number of conserved matches divided by the total number of non-redundant genes in the blocks; tandem duplicated genes were collapsed to a single homolog) were selected as real syntenic regions. For microsynteny analysis, we manually broke the blocks if *At *homologs of independent *Br *sequences in the syntenic blocks were separated by more than 10 kbp. The synteny display is available online at the URL cited in the 'Data used in this study' section.

### Ks analysis of homologous sequences

The timing of duplication events and the divergence of homologous segments was estimated by calculating the number of synonymous substitutions per synonymous site (Ks) between homologous genes. For the model genome comparisons, annotated gene models were used, whereas for the comparison between the *Brassica *genomes, ESTs were analyzed, even though they are error-prone. One drawback associated with the analysis of paralogs derived from ESTs is that multiple entries for the same gene can be included in the dataset, leading to overestimation of redundant Ks measures [5]. However, it is reasonable to assume that redundant Ks measures are randomly distributed among all the Ks values; thus. the effect of redundancy is likely to have been neutral. Before comparing the Ks distribution for EST paralogs and genome sequences of *Br*, we carefully checked the patterns of Ks in the EST data; we did not find any significantly overestimated bulges or peaks. To identify orthologs and paralogs, the protein sequences of the gene models or ESTs were aligned using the all-against-all alignment and the resulting alignment was used as a reference to align the nucleotide sequences. After removing gaps, the Ks values from pairwise alignments of homologous sequences were determined using the maximum likelihood method implemented in the CODEML [70] program of the PAML [71] package under the F3×4 model, similar to the analysis described by Blanc *et al*. [25]. We compared the mode rather than the mean of Ks distributions, because the mode is not affected by bias due to incorrectly defined homolog pairs, which is partly responsible for unexpected overestimation of Ks. Only gene pairs with a Ks estimate of <5 were considered for further evaluation and their Ks age distribution was calculated using the interval 0.02 to 0.1. Divergence time calculations were based on the neutral substitution rate of 1.5 × 10^-8 ^substitutions per site per year for chalcone synthase (*Chs*) and alcohol dehydrogenase (*Adh*) [40].

### Gene conservation between sister blocks in the *B. rapa *genome

Because *Br *BAC clones were selected to minimally overlap the target *At *region, self comparison of *Br *sequences using the DiagHunter program found few duplicated regions (Figure S4 in Additional data file 2). Instead, we manually identified sister blocks of duplication events by using synteny group information between *Br *and *At*. *Br *sequence blocks were defined as putative sister blocks of 3R if two different sequence blocks showed high synteny with respect to *At *regions known to be duplicated remnants of 3R [72], whereas independent *Br *sequence blocks sharing the same syntenic relationship with a single *At *region were selected as sister blocks of 4R. For additional validation, we compared the Ks distribution modes between the paralog gene pairs in the sister blocks.

### Data used in this study

All the data used in this study can be accessed online at [73].

## Abbreviations

*AK*: ancestral karyotype; *At*: *Arabidopsis thaliana*; BAC: bacterial artificial chromosome; *Bc*: *Brassica carinata*; BES: BAC end sequence; *Bj*: *Brassica juncea*; *Bn*: *Brassica nigra*; *Bna*: *Brassica napus*; *Bo*: *Brassica oleracea*; *Br*: *Brassica rapa*; EST: expressed sequence tag; FISH: fluorescent *in situ *hybridization; kbp, kilobase-pairs; Ks: substitutions per synonymous site; LTR: long terminal repeat retrotransposon; Mbp: megabase-pair; *Mt*: *Medicago truncatula*; MYA: million years ago; NCBI: National Center for Biotechnology Information; *Os*: *Oryza sativa*; *Pt*: *Populus trichocarpa*; TE: transposable element; WGD: whole genome duplication; WGT: whole genome triplication.

## Authors' contributions

JHM designed research, performed the experiments, analyzed data, and wrote the manuscript. SJK and TJY designed research and contributed analytic tools. SJK, MJ, JAK, MHL, JSK, KBL, and SIL contributed to data acquisition. YJS and SB developed the database and interfaces to display results on the web. SB, BSC, HJY, DSK, NK, and JHH analyzed data. HJY, IB, and YPL participated in manuscript preparation. BSP conceived the project and supervised its execution.

## Additional data files

The following additional data are available with the online version of this paper: Tables S1, S2, S3, S4, S5 and S6 (Additional file 1); Figures S1, S2, S3 and S4 (Additional file 2); a spreadsheets listing synteny blocks between *Br *and *At *genomes (Additional file 3); spreadsheets describing genome blocks and block boundaries of the ancestral karyotype (*AK*) mapped on the *B. rapa *chromosomes based on *Br*-*At *synteny and *At*-*AK *correspondences (Additional file 4).

## Supplementary Material

Additional data file 1 Table S1: summary of *B. rapa *sequence contigs, constituent BAC associations, and targeting of homologous regions of *A. thaliana *based on BLASTZ matches. Table S2: location of sequence contigs on the *B. rapa *chromosomes according to a combination of genetic map position, FISH results, physical map contig, and positional information from *A. thaliana *counterparts. Table S3: statistics of microsynteny in the synteny blocks identified by a genome comparison of *B. rapa *and *A. thaliana*. Table S4: identification of sister blocks produced by the same polyploidy events in the *Br *genome based on *At*-*At *and *At*-*Br *relationships. Table S5: identification of sister blocks produced by the same polyploidy events in the *Bo *genome. Table S6: sources of genomic and transcript sequences used in this study. Click here for file

Additional data file 2 Figure S1: comparison of homologous block end-point distances between 410 *B. rapa *sequence contigs and their *Arabidopsis *counterpart regions, indicating a genome shrinkage of approximately 30% in *B. rapa*. Figure S2: abundance of different transposable element types in the *B. rapa *genome. Figure S3: comparison of *Brassica *'A' genome structures between *B. rapa *and *B. napus*. Genome blocks were defined based on 24 *AK *genome building blocks. The genome structure of *Bna *was obtained from the reports of Parkin *et al*. [23] and Schranz *et al*. [37]. Regions characterized by significant rearrangements (pink box) or insertions/deletions (gray box) between genomes are highlighted by colored boxes. Scale bars on the margins indicate megabase-pairs (Mbp) for *Br *or centi-Morgans (cM) for *Bna*. Blocks with the opposite orientation relative to *AK *are indicated by a gray upward-pointing arrow on the right side of the block. Figure S4: dot plot of *B. rapa *compared with itself. Each dot in the dot plot represents a reciprocal best BLASTP match between gene pairs at a cutoff value of <E^-20^. Red dots show the regions of synteny identified by DiagHunter. Click here for file

Additional data file 3 The data provided include two worksheets. Gene correspondence data for the 227 synteny blocks identified by DiagHunter are provided in the worksheet entitled 'blocks.' The synteny quality of the blocks is included in the worksheet named 'quality'; tandem duplicated genes were considered to be single homologs. The quality of synteny was defined as described in the Materials and methods. Click here for file

Additional data file 4 This dataset indicates the block order, orientation, and color-coding used for each *AK *block. The synteny-defined block boundaries on the *Br *chromosomes identified by DiagHunter were defined according to the *At *locus name described by Schranz *et al*. [37]. Supporting data, including gene correspondences in all synteny blocks, are included in the worksheet 'At-Br matches in synteny blocks'. Mapping of *AK *blocks to the *Br *chromosomes, including block boundaries and orientation, is provided in the worksheet '*AK *blocks to *Br*'. A summary table and graph of *AK *mapping to the *Br *chromosomes is provided in the worksheet 'Br chr. chart'. Click here for file
