# Supplementary material for: Genome-wide comparative analysis of the Brassica rapa gene space reveals genome shrinkage and differential loss of duplicated genes after whole genome triplication
Source: Genome Biol. 2009 Oct 12;10(10):R111. doi: 10.1186/gb-2009-10-10-r111 (PMC2784326; doi:10.1186/gb-2009-10-10-r111)
Supplement: Additional data file 1 — Table S1: summary of B. rapa sequence contigs, constituent BAC associations, and targeting of homologous regions of A. thaliana based on BLASTZ matches. Table S2: location of sequence contigs on the B. rapa chromosomes according to a combination of genetic map position, FISH results, physical map contig, and positional information from A. thaliana counterparts. Table S3: statistics of microsynteny in the synteny blocks identified by a genome comparison of B. rapa and A. thaliana. Table S4: identification of sister blocks produced by the same polyploidy events in the Br genome based on At-At and At-Br relationships. Table S5: identification of sister blocks produced by the same polyploidy events in the Bo genome. Table S6: sources of genomic and transcript sequences used in this study. [file gb-2009-10-10-r111-S1.PDF]

Table S1. Summary of *B. rapa* sequence contigs, constituent BAC associations, and targeting of homologous regions of *A. thaliana* based on BLASTZ matches.

Brassica rapa					*Arabidopsis thaliana*			
Contig	Length (bp)	Constituent BACs	Accession	Phase	Chromosome	Start position	End position	Length (bp)
001	114,789	KBrB063A04	AC232510	2	At1	31,237	166,379	135,142
002	77,450	KBrB046G18	AC189365	2	At1	102,555	228,041	125,486
003	583,577	KBrB092L06	AC189527	2	At1	448,546	1,514,046	1,065,500
		KBrB005I07	AC232441	2				
		KBrB090K20	AC232533	3				
		KBrB089B13	AC189509	3				
		KBrB043F18	AC189351	2				
		KBrB002B23	AC232437	3				
		KBrB017P15	AC189246	3				
004	132,571	KBrB030F10	AC189302	3	At1	624,039	753,147	129,108
005	144,870	KBrB013C03	AC189229	3	At1	1,510,278	1,854,223	343,945
006	144,320	KBrH053G06	AC189609	2	At1	1,908,489	1,992,231	83,742
007	159,921	KBrB060D14	AC189410	1	At1	1,979,125	2,081,656	102,531
008	168,997	KBrB012F17	AC189226	2	At1	2,085,721	2,281,627	195,906
009	137,645	KBrH138P04	AC155338	3	At1	2,125,327	2,302,896	177,569
010	100,361	KBrH034K06	AC232558	2	At1	2,330,528	2,547,586	217,058
011	104,777	KBrB090M17	AC189518	3	At1	2,421,409	2,620,403	198,994
012	240,940	KBrH001D23	AC189533	2	At1	2,578,342	3,073,053	494,711
		KBrH006H02	AC232544	2				
013	117,645	KBrH013B19	AC189590	2	At1	2,818,630	3,009,625	190,995
014	202,737	KBrB037O12	AC189331	3	At1	2,994,364	3,337,521	343,157
		KBrB091G04	AC189519	3				
015	110,739	KBrS004C14	AC232571	2	At1	3,281,652	3,473,638	191,986
016	128,804	KBrB086J10	AC172864	2	At1	3,483,323	3,608,103	124,780
017	247,720	KBrB006B05	AC232443	2	At1	4,208,805	4,754,593	545,788
		KBrB017B11	AC172858	3				
018	99,248	KBrS016D08	AC232579	2	At1	4,444,141	4,514,309	70,168
019	201,056	KBrB070M24	AC172863	3	At1	4,739,699	4,960,978	221,279
		KBrH010P01	AC232549	2				
020	131,149	KBrB086M23	AC189499	3	At1	4,955,927	5,077,020	121,093
021	109,322	KBrB090H23	AC232532	2	At1	5,064,678	5,164,600	99,922
022	100,775	KBrB056G18	AC232502	2	At1	5,132,667	5,236,043	103,376
023	87,479	KBrB034J13	AC232477	1	At1	5,231,475	5,306,884	75,409
024	105,620	KBrB077F22	AC189466	2	At1	5,300,862	5,719,276	418,414
025	146,230	KBrB041H16	AC232483	2	At1	5,687,229	5,839,801	152,572
		KBrB049E19	AC172862	2				
026	116,463	KBrS004B22	AC172887	3	At1	5,795,958	6,032,436	236,478
027	116,291	KBrH014P02	AC172875	3	At1	6,154,635	6,284,133	129,498
028	128,505	KBrH092O19	AC232564	2	At1	6,187,585	6,424,882	237,297
029	123,397	KBrB044C04	AC172861	2	At1	6,484,158	6,668,815	184,657
030	388,205	KBrS007I12	AC172888	2	At1	6,628,911	6,859,002	230,091
		KBrB043O20	AC189356	3				
		KBrH006P24	AC189558	3				
031	137,362	KBrB058M10	AC189405	3	At1	6,883,693	7,158,620	274,927
		KBrB042N05	AC189346	3				
032	326,661	KBrB018K09	AC189247	3	At1	7,150,534	7,387,905	237,371
		KBrB011L09	AC189224	3				

		KBrB076B03	AC189465	3				
033	161,929	KBrB019O05	AC189254	2	At1	7,400,068	7,607,484	207,416
034	169,091	KBrB023N08	AC189273	2	At1	7,595,068	7,891,345	296,277
		KBrB023L08	AC189271	3				
035	136,069	KBrH003E21	AC189538	3	At1	7,847,446	7,929,911	82,465
036	240,690	KBrH020D15	AC155340	3	At1	8,047,504	8,250,790	203,286
		KBrB048C04	AC189371	3				
037	137,197	KBrB006C05	AC232444	2	At1	8,216,008	8,434,924	218,916
038	145,633	KBrB080N15	AC189476	3	At1	8,853,999	8,998,123	144,124
039	325,515	KBrB089L03	AC189513	2	At1	8,995,173	9,304,219	309,046
		KBrB066M16	AC189429	3				
		KBrB059J21	AC189408	2				
040	113,160	KBrB052E19	AC189385	3	At1	9,274,520	9,422,091	147,571
041	138,607	KBrB024J13	AC189274	2	At1	9,375,059	9,426,640	51,581
042	78,427	KBrS010I09	AC189649	3	At1	9,493,340	9,577,271	83,931
043	164,862	KBrH006E24	AC189555	3	At1	9,540,160	9,789,975	249,815
		KBrB003O07	AC189194	3				
044	109,380	KBrB025C06	AC232468	2	At1	9,911,938	10,031,090	119,152
045	320,040	KBrB016E20	AC189240	2	At1	10,035,138	10,553,458	518,320
		KBrH012O06	AC189589	2				
		KBrB046G20	AC189366	3				
046	44,744	KBrB027K16	AC189296	3	At1	10,655,250	10,674,666	19,416
047	127,059	KBrB042D24	AC189342	3	At1	10,728,983	10,819,265	90,282
048	92,260	KBrS003D10	AC189628	3	At1	10,779,364	10,896,101	116,737
049	190,239	KBrB053L06	AC189390	3	At1	10,936,911	11,088,550	151,639
		KBrB012O24	AC189228	2				
050	275,300	KBrH089C20	AC232590	2	At1	11,046,912	11,222,416	175,504
		KBrB016K20	AC189241	2				
		KBrB070J23	AC189444	3				
051	109,512	KBrB074J19	AC189462	2	At1	11,254,228	11,276,095	21,867
052	138,010	KBrB026E21	AC232470	2	At1	11,394,679	11,470,983	76,304
053	159,384	KBrB080J22	AC189475	3	At1	11,470,259	11,560,904	90,645
054	135,829	KBrB019O20	AC189255	3	At1	11,655,771	11,773,532	117,761
055	139,834	KBrB041L12	AC189341	3	At1	12,127,931	12,217,449	89,518
056	132,964	KBrB065E07	AC232513	3	At1	12,355,953	12,655,972	300,019
057	125,570	KBrH004M10	AC189546	3	At1	17,569,144	17,729,692	160,548
058	115,119	KBrH080L24	AC155345	3	At1	17,700,798	17,720,627	19,829
059	107,617	KBrB049A09	AC232490	3	At1	18,039,565	18,156,607	117,042
060	165,235	KBrH012G24	AC189585	2	At1	18,105,624	18,266,210	160,586
061	132,760	KBrB065N20	AC189427	3	At1	18,514,338	18,660,591	146,253
062	137,375	KBrB030D08	AC189301	3	At1	18,665,629	18,865,779	200,150
		KBrH109M07	AC232567	2				
063	130,699	KBrB018N18	AC189248	3	At1	19,194,382	19,415,194	220,812
064	204,490	KBrB022P06	AC189268	3	At1	19,525,342	19,795,407	270,065
		KBrS012H18	AC189654	2				
065	99,686	KBrH003K23	AC189539	2	At1	20,189,187	20,587,238	398,051
066	133,598	KBrH013K13	AC189592	3	At1	20,599,154	20,722,124	122,970
067	119,683	KBrH001D10	AC189531	2	At1	20,712,120	20,883,081	170,961
068	130,819	KBrB086C10	AC189496	3	At1	21,132,535	21,262,720	130,185
069	108,171	KBrB037F09	AC189330	3	At1	21,493,016	21,504,680	11,664
070	148,559	KBrB036K20	AC189322	3	At1	21,971,630	22,131,759	160,129
071	123,455	KBrB092B15	AC189524	3	At1	22,561,170	22,704,766	143,596

072	159,334	KBrB069A23	AC189437	3	At1	23,375,273	23,524,264	148,991
073	137,649	KBrB025K04	AC189288	3	At1	23,529,980	23,653,002	123,022
074	123,278	KBrH092K14	AC232563	2	At1	23,559,081	23,653,002	93,921
075	104,182	KBrB022A09	AC189264	3	At1	23,689,065	23,827,162	138,097
076	146,304	KBrB070J11	AC189443	3	At1	24,178,632	24,339,139	160,507
077	112,576	KBrB083K19	AC189484	3	At1	24,336,204	24,556,589	220,385
078	114,731	KBrB010O09	AC189223	3	At1	24,624,596	24,752,376	127,780
079	143,202	KBrB027F23	AC232473	2	At1	24,919,763	25,195,085	275,322
080	135,708	KBrB005J17	AC189200	3	At1	25,086,237	25,218,162	131,925
081	111,705	KBrH010F15	AC189572	3	At1	25,209,324	25,371,765	162,441
082	289,875	KBrB008G18	AC232449	3	At1	25,377,809	25,688,010	310,201
		KBrB084K02	AC189489	3				
083	103,293	KBrB034A02	AC189308	3	At1	25,717,571	25,833,399	115,828
084	102,870	KBrB026C23	AC189291	3	At1	25,760,301	25,941,449	181,148
085	338,361	KBrB073F16	AC189458	3	At1	25,921,972	26,220,301	298,329
		KBrB028I01	AC189298	3				
		KBrH012E04	AC189584	3				
		KBrB043L15	AC189353	3				
086	116,983	KBrB064F02	AC232511	1	At1	26,259,002	26,352,150	93,148
087	103,407	KBrB073K15	AC189459	2	At1	26,334,322	26,474,804	140,482
088	112,903	KBrB034I14	AC189311	3	At1	26,485,125	26,603,147	118,022
089	129,699	KBrB084M08	AC189492	3	At1	26,601,905	26,710,275	108,370
090	147,020	KBrB057E05	AC189401	3	At1	26,625,149	26,915,527	290,378
091	132,391	KBrB055K07	AC189395	2	At1	26,907,890	27,026,563	118,673
092	213,044	KBrB038O16	AC189334	3	At1	27,025,239	27,354,025	328,786
		KBrH011C10	AC189575	3				
093	96,143	KBrB088E11	AC189507	3	At1	27,342,093	27,450,580	108,487
094	144,247	KBrB086N06	AC189500	3	At1	27,450,228	27,610,965	160,737
095	121,782	KBrH014G16	AC189598	2	At1	27,579,556	27,789,405	209,849
096	117,834	KBrB056L15	AC189400	3	At1	27,776,419	27,896,101	119,682
097	129,514	KBrH011B08	AC189574	3	At1	27,875,667	28,013,097	137,430
098	109,823	KBrH015N11	AC189605	3	At1	27,990,760	28,149,736	158,976
099	107,138	KBrB074J14	AC189461	2	At1	28,103,624	28,205,246	101,622
100	212,667	KBrB084P16	AC189493	3	At1	28,197,092	28,409,157	212,065
		KBrH012I05	AC189586	3				
101	278,307	KBrB026G01	AC189295	3	At1	28,377,740	29,072,827	695,087
		KBrB041J04	AC189339	3				
		KBrB006H21	AC189203	3				
102	410,639	KBrB021P15	AC189262	2	At1	29,017,585	29,469,444	451,859
		KBrB047M06	AC189370	3				
		KBrB061K11	AC189414	3				
		KBrB068K17	AC189435	3				
103	136,510	KBrB006F18	AC232445	3	At1	29,467,089	29,801,678	334,589
104	216,901	KBrB007M04	AC189209	3	At1	29,753,538	30,000,682	247,144
		KBrB034L08	AC189312	3				
105	304,869	KBrB026K21	AC232471	3	At1	30,024,834	30,406,480	381,646
		KBrB044D01	AC232487	3				
		KBrB034F07	AC232476	3				
106	152,733	KBrB051H12	AC189382	2	At2	170,829	285,026	114,197
107	142,422	KBrB008C11	AC189211	2	At2	429,541	552,369	122,828
108	153,081	KBrB072E02	AC189452	3	At2	556,557	959,108	402,551
		KBrH006N19	AC189556	2				

109	93,865	KBrH009H15	AC189567	3		At2	867,367	1,031,444	164,077
110	131,452	KBrB010H08	AC232451	2		At2	1,149,337	1,286,020	136,683
111	323,857	KBrB058B22	AC189403	2		At2	1,581,646	2,183,883	602,237
		KBrS008C11	AC189641	3					
		KBrB011D06	AC232452	2					
		KBrB037E22	AC232481	2					
112	168,348	KBrH001K17	AC232537	3		At2	6,315,474	6,519,481	204,007
		KBrB091E13	AC232534	3					
113	128,451	KBrB057N22	AC232504	3		At2	6,913,625	7,129,297	215,672
114	91,499	KBrH006K14	AC232545	2		At2	7,217,033	7,497,459	280,426
115	112,002	KBrB023O12	AC232465	2		At2	7,571,972	7,658,534	86,562
116	207,689	KBrH004A02	AC232540	3		At2	7,700,726	7,845,353	144,627
		KBrB024N11	AC232467	3					
117	168,010	KBrB059K16	AC232506	2		At2	7,804,284	7,988,188	183,904
		KBrH011J16	AC232550	3					
118	125,950	KBrB022C05	AC189265	1		At2	8,467,325	8,513,989	46,664
119	128,084	KBrH009P18	AC189570	3		At2	8,505,583	8,748,385	242,802
120	131,375	KBrH001J06	AC189534	3		At2	8,729,574	8,777,026	47,452
121	718,461	KBrH095F22	AC232565	2		At2	8,747,195	9,518,967	771,772
		KBrB017F11	AC189244	2					
		KBrB082F21	AC189480	3					
		KBrH081N08	AC155346	3					
		KBrB019I24	AC189251	2					
		KBrB025M01	AC189289	3					
		KBrS009B06	AC232574	2					
		KBrB035J16	AC232478	2					
		KBrH070I10	AC172879	3					
122	97,649	KBrH014M07	AC189600	2		At2	8,751,484	8,845,860	94,376
123	102,286	KBrB043N10	AC232485	2		At2	9,852,483	9,996,242	143,759
124	47,472	KBrH001N23	AC232538	2		At2	10,075,679	10,112,467	36,788
125	139,805	KBrB038O14	AC232482	2		At2	10,483,714	10,691,816	208,102
126	251,260	KBrS008H22	AC189643	2		At2	10,683,946	11,113,729	429,783
		KBrB063K02	AC189420	2					
127	150,947	KBrB060F02	AC189412	3		At2	11,056,282	11,544,701	488,419
		KBrH010F07	AC189571	3					
128	124,756	KBrB038M13	AC189333	3		At2	11,436,264	11,762,408	326,144
129	193,890	KBrB014N06	AC189236	2		At2	11,747,884	11,968,358	220,474
		KBrS006L21	AC189640	3					
130	44,927	KBrB019J14	AC232458	3		At2	11,964,165	12,020,610	56,445
131	118,051	KBrB028K05	AC189299	3		At2	12,133,929	12,230,279	96,350
132	105,441	KBrB068E07	AC189432	3		At2	12,216,771	12,627,902	411,131
133	125,891	KBrS012H21	AC232577	3		At2	12,675,449	12,777,366	101,917
134	887,770	KBrH038M21	AC232559	3		At2	12,802,153	14,060,783	1,258,630
		KBrB026F03	AC189293	2					
		KBrB071A06	AC232518	2					
		KBrH005J02	AC189551	3					
		KBrB068H20	AC189433	3					
		KBrB074K06	AC189463	3					
		KBrH003D18	AC189536	3					
		KBrB082C18	AC232525	2					
		KBrB080O07	AC189477	2					
135	141,310	KBrB084C18	AC189485	3		At2	13,813,476	13,949,978	136,502

136	137,894	KBrB052E10	AC189384	3	At2	14,074,943	14,131,503	56,560
137	199,743	KBrS011C02	AC189651	3	At2	14,084,258	14,293,678	209,420
		KBrB026E08	AC189292	3				
138	151,196	KBrB047H21	AC232488	2	At2	14,223,930	14,525,726	301,796
139	194,188	KBrS012M03	AC189655	3	At2	14,563,971	14,938,374	374,403
		KBrB042N23	AC189347	1				
140	138,876	KBrB089M13	AC189515	2	At2	14,922,715	15,061,692	138,977
141	222,595	KBrB050K06	AC189380	3	At2	15,010,714	15,218,310	207,596
		KBrS010A05	AC232575	3				
142	105,141	KBrB031G07	AC189304	2	At2	15,183,632	15,329,601	145,969
143	129,760	KBrS003G14	AC189630	3	At2	15,326,352	15,534,179	207,827
144	116,270	KBrH007P05	AC189561	3	At2	15,506,991	15,642,630	135,639
145	95,655	KBrH015L23	AC232555	2	At2	15,588,469	15,694,430	105,961
146	110,106	KBrB082L07	AC189482	2	At2	15,689,120	15,773,066	83,946
147	196,173	KBrB033O04	AC189307	3	At2	15,769,349	15,952,731	183,382
		KBrB089D07	AC189510	3				
148	234,425	KBrH004D08	AC189543	3	At2	15,969,610	16,254,366	284,756
		KBrB002P20	AC189192	3				
149	305,614	KBrB023F24	AC189270	2	At2	16,222,045	16,565,145	343,100
		KBrB051I12	AC189383	2				
150	104,954	KBrB032C14	AC189306	3	At2	16,536,181	16,706,914	170,733
151	121,062	KBrS005G02	AC232572	3	At2	16,679,924	16,825,168	145,244
152	216,654	KBrB092D10	AC189525	1	At2	16,810,135	16,944,672	134,537
		KBrH014A02	AC189596	3				
153	129,808	KBrH012A14	AC189581	1	At2	16,913,963	17,160,444	246,481
154	122,818	KBrB086M08	AC232531	3	At2	17,156,790	17,316,783	159,993
155	162,363	KBrH009I04	AC189568	3	At2	17,258,946	17,498,961	240,015
156	279,432	KBrH117N09	AC155337	3	At2	17,476,767	17,765,476	288,709
		KBrB010H06	AC189220	3				
		KBrH012N11	AC189588	3				
157	111,776	KBrB014C24	AC189234	2	At2	17,849,042	17,954,664	105,622
158	157,957	KBrH014M21	AC189601	2	At2	17,947,962	18,102,484	154,522
159	119,637	KBrB056I08	AC189399	3	At2	18,321,704	18,625,954	304,250
160	41,938	KBrB016N13	AC189242	3	At2	18,651,165	18,694,950	43,785
161	150,900	KBrB086L12	AC189498	3	At2	18,685,009	18,809,342	124,333
162	142,879	KBrB073H13	AC232521	2	At2	18,731,468	18,926,036	194,568
163	252,785	KBrH011O17	AC189580	3	At2	18,824,314	19,409,344	585,030
		KBrB050C17	AC232492	2				
		KBrB049N04	AC232491	3				
164	308,529	KBrB061N03	AC189416	3	At2	19,400,748	19,688,436	287,688
		KBrB042E01	AC232484	2				
		KBrS004I08	AC189634	3				
165	111,800	KBrB056F17	AC232501	2	At3	8,724	74,005	65,281
166	191,134	KBrB049D17	AC189377	2	At3	28,394	337,524	309,130
		KBrB055E21	AC232500	2				
167	135,946	KBrB020F06	AC232459	3	At3	363,958	466,087	102,129
168	73,024	KBrB024M19	AC232466	2	At3	363,958	441,040	77,082
169	302,431	KBrS007P17	AC232573	2	At3	399,330	1,196,133	796,803
		KBrB001H24	AC189185	3				
		KBrB058F21	AC189404	3				
170	66,709	KBrB078A03	AC189468	3	At3	1,119,931	1,256,622	136,691
171	172,725	KBrB043L02	AC189352	2	At3	1,259,233	1,513,414	254,181

		KBrB037C07	AC189329	3					
172	84,700	KBrH012A23	AC189582	2		At3	1,488,871	1,659,090	170,219
173	443,146	KBrB003A10	AC189193	3		At3	1,618,909	2,242,276	623,367
		KBrH013F17	AC232553	2					
		KBrB013O20	AC189233	3					
		KBrH005H18	AC189550	3					
174	121,613	KBrH011G10	AC189577	3		At3	2,241,452	2,438,892	197,440
175	297,862	KBrB042L19	AC189345	2		At3	2,388,020	2,671,655	283,635
		KBrB072L17	AC189454	3					
		KBrB047A09	AC189368	3					
176	109,674	KBrB055G10	AC189394	3		At3	2,823,519	3,007,826	184,307
177	132,619	KBrH003N18	AC189540	3		At3	3,007,564	3,224,660	217,096
178	146,314	KBrB087P06	AC189504	1		At3	3,206,586	3,360,332	153,746
179	145,501	KBrB068B07	AC189431	3		At3	3,332,745	3,560,299	227,554
180	153,826	KBrH004P05	AC189547	3		At3	3,557,024	3,689,492	132,468
181	141,404	KBrH122D17	AC232569	2		At3	3,633,800	3,871,449	237,649
182	369,463	KBrB063G23	AC189419	2		At3	3,860,353	4,300,419	440,066
		KBrB039A19	AC189335	3					
		KBrB069G15	AC189439	2					
183	156,768	KBrB053K16	AC232497	2		At3	4,209,634	4,368,149	158,515
184	431,628	KBrB047D06	AC189369	3		At3	4,356,247	5,037,685	681,438
		KBrH001D20	AC189532	2					
		KBrB019N06	AC189253	3					
		KBrB042O05	AC189349	2					
185	137,428	KBrH001C24	AC189530	3		At3	5,027,750	5,133,999	106,249
186	107,372	KBrB021F10	AC189256	2		At3	5,090,380	5,273,179	182,799
187	107,263	KBrB021B05	AC232460	3		At3	5,267,088	5,342,493	75,405
188	119,016	KBrB089M05	AC189514	2		At3	5,296,316	5,398,899	102,583
189	271,435	KBrB010F13	AC189218	3		At3	5,371,430	5,796,685	425,255
		KBrB052N08	AC189387	2					
		KBrB036B21	AC189317	3					
190	127,874	KBrB042O03	AC189348	3		At3	5,775,290	5,892,325	117,035
191	103,160	KBrH013M23	AC189593	3		At3	5,845,360	5,940,753	95,393
192	225,613	KBrB019B16	AC189250	2		At3	6,031,185	6,428,658	397,473
		KBrB070K15	AC232517	2					
193	138,301	KBrB071H12	AC189447	3		At3	6,434,763	6,508,753	73,990
194	136,873	KBrH054E04	AC232562	3		At3	6,496,206	6,599,020	102,814
195	108,401	KBrB077L14	AC189467	3		At3	6,632,413	6,683,192	50,779
196	139,941	KBrB082I19	AC189481	2		At3	6,681,883	6,785,062	103,179
197	111,492	KBrB017O23	AC232453	2		At3	6,782,832	6,837,517	54,685
198	133,781	KBrB044L16	AC189359	3		At3	6,840,937	7,061,195	220,258
199	161,318	KBrB004O19	AC189198	2		At3	7,050,673	7,139,490	88,817
200	90,316	KBrB045I12	AC189361	3		At3	7,089,206	7,189,368	100,162
201	141,898	KBrB009B09	AC189213	2		At3	7,189,135	7,451,659	262,524
202	102,122	KBrB042J11	AC189344	3		At3	7,399,178	7,475,302	76,124
203	229,485	KBrB092B03	AC189522	2		At3	7,475,296	7,627,760	152,464
		KBrB046C11	AC189364	3					
204	138,519	KBrB059A03	AC189406	2		At3	7,604,138	7,818,240	214,102
205	240,905	KBrS005O12	AC189639	2		At3	7,835,162	7,956,875	121,713
		KBrB039P21	AC189337	2					
206	101,150	KBrB037A01	AC189327	3		At3	7,914,810	8,108,334	193,524
207	103,923	KBrB072L19	AC189455	2		At3	8,113,223	8,161,031	47,808

208	200,410	KBrB026F22	AC189294	2	At3	8,140,042	8,708,225	568,183
		KBrB073E20	AC232520	2				
209	134,297	KBrB048K11	AC189374	2	At3	8,679,184	8,732,355	53,171
210	141,878	KBrB084M06	AC189491	3	At3	8,726,428	8,906,040	179,612
211	101,717	KBrB072K21	AC232519	3	At3	8,999,019	9,155,094	156,075
212	140,401	KBrB008D15	AC232448	3	At3	9,303,078	9,388,602	85,524
213	125,039	KBrH010N11	AC189573	3	At3	9,460,772	9,713,516	252,744
		KBrB007A19	AC189206	3				
214	96,532	KBrB090B08	AC189516	2	At3	9,702,439	9,801,277	98,838
215	130,038	KBrB089H11	AC189512	2	At3	9,752,271	9,939,295	187,024
216	125,183	KBrB004L01	AC232440	2	At3	9,822,560	10,029,091	206,531
217	214,057	KBrH102C07	AC232566	2	At3	9,953,930	10,687,423	733,493
		KBrH123C19	AC232591	2				
218	132,599	KBrS004A14	AC189633	3	At3	10,252,668	10,446,543	193,875
219	154,876	KBrH006L19	AC172871	3	At3	10,502,551	10,850,227	347,676
220	140,130	KBrB027J02	AC232587	2	At3	10,789,218	11,076,897	287,679
221	74,351	KBrH013N10	AC189594	3	At3	10,941,793	11,137,871	196,078
222	110,359	KBrH014A01	AC189595	2	At3	11,047,187	11,295,128	247,941
223	122,391	KBrS011C11	AC232592	2	At3	17,525,154	17,709,961	184,807
224	260,371	KBrB044D19	AC189357	3	At3	18,159,673	18,328,481	168,808
		KBrB062J16	AC189417	3				
225	146,490	KBrB051E13	AC189381	2	At3	18,317,038	18,422,496	105,458
226	137,318	KBrB013J16	AC189230	2	At3	18,430,289	18,616,070	185,781
		KBrB043L22	AC189354	2				
227	124,005	KBrB049N17	AC189379	3	At3	18,677,362	18,775,944	98,582
228	121,528	KBrB002E24	AC189189	3	At3	18,866,027	18,929,517	63,490
229	97,596	KBrB088I08	AC189508	3	At3	18,929,416	19,000,745	71,329
230	348,421	KBrS001M03	AC189627	3	At3	18,956,714	19,477,537	520,823
		KBrS012D09	AC189652	2				
		KBrB084F01	AC189486	2				
231	134,403	KBrB063M21	AC189422	3	At3	19,477,022	19,581,652	104,630
232	215,000	KBrB087M20	AC189502	3	At3	19,636,956	19,817,563	180,607
		KBrB023B16	AC189269	3				
233	271,953	KBrB023N04	AC189272	2	At3	19,858,026	20,313,959	455,933
		KBrB048D12	AC189372	2				
234	281,193	KBrH012N08	AC232589	2	At3	20,311,130	20,490,523	179,393
		KBrH117O12	AC189620	2				
		KBrS001F19	AC189625	2				
235	159,621	KBrB084F02	AC189487	2	At3	20,487,724	20,616,681	128,957
236	126,999	KBrB056C05	AC189397	3	At3	20,616,574	20,693,445	76,871
237	151,767	KBrB084H08	AC189488	2	At3	20,702,640	20,954,147	251,507
238	129,757	KBrB073D09	AC189456	3	At3	20,937,927	21,062,348	124,421
239	102,939	KBrB088E10	AC189506	3	At3	21,047,155	21,162,252	115,097
240	230,097	KBrB013M01	AC189231	3	At3	21,153,305	21,353,061	199,756
		KBrB073E19	AC189457	3				
241	127,990	KBrB078G17	AC189469	3	At3	21,343,627	21,469,580	125,953
242	121,934	KBrB070L01	AC189445	3	At3	21,453,642	21,605,399	151,757
243	156,080	KBrB010L05	AC189221	3	At3	21,565,025	21,750,864	185,839
244	116,392	KBrS009G24	AC189647	3	At3	21,746,995	21,944,467	197,472
245	211,829	KBrB081E09	AC189478	2	At3	21,896,420	22,132,746	236,326
		KBrB070F08	AC232588	2				
246	149,009	KBrB027O09	AC189297	3	At3	22,021,638	22,349,030	327,392

247	171,751	KBrB036H01	AC189319	3	At3	22,343,021	22,514,217	171,196
		KBrB021E01	AC232461	2				
248	137,515	KBrB025O08	AC189290	1	At3	22,484,734	22,784,532	299,798
249	216,104	KBrB044E18	AC189358	3	At3	22,732,496	22,915,902	183,406
		KBrH012J09	AC232551	2				
250	136,781	KBrH007M24	AC189560	2	At3	22,789,915	22,892,689	102,774
251	143,428	KBrH110N19	AC189619	2	At3	22,997,995	23,193,178	195,183
252	121,576	KBrS008K03	AC189644	2	At3	23,246,113	23,329,630	83,517
253	93,030	KBrB071M14	AC189449	3	At3	23,328,892	23,405,716	76,824
		KBrB065N19	AC189426	2				
254	129,754	KBrB041F06	AC189338	2	At4	151,566	326,890	175,324
255	105,785	KBrB037A09	AC189328	2	At4	447,118	536,508	89,390
256	137,666	KBrH121P05	AC189621	3	At4	741,285	927,851	186,566
257	222,948	KBrB027P21	AC232474	2	At4	1,064,350	1,241,141	176,791
		KBrH129I15	AC232570	2				
258	124,735	KBrB018D16	AC232454	3	At4	1,108,500	1,308,779	200,279
259	132,867	KBrB002P01	AC232438	3	At4	1,332,080	1,525,404	193,324
260	101,772	KBrB037E04	AC232480	3	At4	1,497,492	1,607,711	110,219
261	105,749	KBrB021M05	AC189259	2	At4	2,325,516	2,433,742	108,226
262	134,601	KBrB061C02	AC232509	3	At4	5,785,078	6,176,960	391,882
263	142,706	KBrB051E01	AC232493	3	At4	6,270,734	6,657,116	386,382
264	143,215	KBrB007I08	AC232447	2	At4	6,609,359	6,804,395	195,036
265	130,486	KBrH009K01	AC232547	3	At4	6,950,272	7,213,014	262,742
266	143,205	KBrB019J08	AC232457	2	At4	7,260,892	7,350,059	89,167
267	124,067	KBrB043N17	AC232486	2	At4	8,003,112	8,050,006	46,894
268	104,786	KBrB074G23	AC189460	2	At4	8,500,108	8,555,202	55,094
269	103,616	KBrB085G17	AC189495	3	At4	8,611,816	8,715,243	103,427
270	140,025	KBrB089H07	AC189511	3	At4	8,696,630	8,909,662	213,032
271	371,043	KBrB047I09	AC232489	2	At4	8,965,126	9,432,371	467,245
		KBrB026A07	AC232469	3				
		KBrS003O10	AC189632	3				
		KBrB084K23	AC189490	2				
272	96,400	KBrB056G23	AC189398	2	At4	9,452,822	9,636,949	184,127
273	117,558	KBrH122A22	AC232568	3	At4	9,580,351	9,721,375	141,024
274	150,083	KBrH138O03	AC225403	2	At4	9,681,508	9,760,826	79,318
275	122,940	KBrH045E23	AC232561	2	At4	9,726,100	9,876,760	150,660
276	132,630	KBrS005I11	AC189638	1	At4	9,778,327	9,889,268	110,941
277	143,164	KBrB080C12	AC189471	2	At4	9,888,280	10,002,373	114,093
278	120,911	KBrS008P19	AC189645	2	At4	9,939,736	10,063,420	123,684
279	244,579	KBrH006A08	AC189553	3	At4	10,055,311	10,314,578	259,267
		KBrB004G11	AC189196	2				
280	233,034	KBrB023N19	AC232464	2	At4	10,283,277	10,500,757	217,480
		KBrB060L22	AC232508	3				
281	147,357	KBrB065H17	AC232514	3	At4	10,455,646	10,590,484	134,838
282	127,483	KBrB085J21	AC232529	1	At4	10,598,343	10,817,097	218,754
283	108,772	KBrB067M19	AC232515	3	At4	10,806,216	10,951,575	145,359
284	101,821	KBrB021L13	AC232462	3	At4	10,897,677	11,018,095	120,418
285	124,081	KBrB085D18	AC232528	2	At4	11,394,747	11,517,701	122,954
286	36,051	KBrB084N21	AC232527	3	At4	11,529,699	11,546,705	17,006
287	164,310	KBrB055A02	AC232499	2	At4	11,580,286	11,725,379	145,093
		KBrB069A15	AC189436	2				
288	262,681	KBrB075L03	AC189464	3	At4	11,709,673	12,049,125	339,452

		KBrB006A15	AC189202	2				
289	102,789	KBrB001M05	AC189187	2	At4	12,053,350	12,173,730	120,380
290	94,098	KBrS011B08	AC189650	2	At4	12,177,875	12,280,349	102,474
291	252,962	KBrB091M11	AC232535	2	At4	12,278,902	12,429,912	151,010
		KBrB053E08	AC232496	2				
292	195,131	KBrH015O11	AC232556	3	At4	12,392,256	12,533,978	141,722
		KBrB042G14	AC189343	2				
293	137,724	KBrH001M22	AC189535	2	At4	12,532,826	12,626,260	93,434
294	60,322	KBrH005A08	AC189548	3	At4	12,591,032	12,701,134	110,102
295	99,603	KBrB059G16	AC189407	3	At4	12,676,966	12,927,749	250,783
296	126,831	KBrB061L05	AC189415	3	At4	12,904,019	12,986,316	82,297
297	109,297	KBrB034C07	AC189309	3	At4	12,986,346	13,151,133	164,787
298	204,296	KBrB045B23	AC189360	3	At4	13,131,259	13,345,983	214,724
		KBrB028P01	AC189300	3				
299	200,421	KBrH012K23	AC189587	3	At4	13,308,723	13,777,829	469,106
		KBrH004I05	AC189544	3				
300	105,067	KBrB014O06	AC189237	3	At4	13,735,685	13,853,801	118,116
301	68,828	KBrH009D02	AC189566	3	At4	13,904,104	13,941,737	37,633
302	235,654	KBrB069M23	AC189441	3	At4	13,936,422	14,084,157	147,735
		KBrB021P17	AC189263	3				
303	104,265	KBrS003F17	AC189629	2	At4	14,053,272	14,158,065	104,793
304	139,290	KBrB006O19	AC189205	2	At4	14,131,340	14,345,007	213,667
305	238,102	KBrH015E17	AC189604	2	At4	14,360,811	14,572,070	211,259
		KBrB070M16	AC189446	3				
306	118,435	KBrH001F03	AC232536	2	At4	14,632,330	14,726,328	93,998
307	107,887	KBrB090J11	AC189517	2	At4	14,672,812	14,830,328	157,516
308	153,048	KBrB080E18	AC189472	2	At4	14,825,787	14,963,233	137,446
309	151,784	KBrB048L11	AC189375	3	At4	14,952,719	15,134,553	181,834
310	234,616	KBrH011G12	AC189578	3	At4	15,127,469	15,384,533	257,064
		KBrB010F06	AC189217	3				
311	108,012	KBrB079E08	AC232524	2	At4	15,419,858	15,590,608	170,750
312	296,200	KBrB035P03	AC232479	3	At4	15,490,841	15,794,981	304,140
		KBrB010F19	AC189219	3				
		KBrB066A08	AC189428	2				
313	306,155	KBrH009C15	AC189565	3	At4	15,794,504	16,147,244	352,740
		KBrB043B23	AC189350	2				
		KBrH029A20	AC232557	2				
314	513,013	KBrS004O21	AC189637	3	At4	16,032,221	16,577,646	545,425
		KBrB052L10	AC189386	3				
		KBrB036M17	AC189325	3				
		KBrB092P05	AC189529	3				
315	163,034	KBrB010M19	AC189222	3	At4	16,567,755	16,740,296	172,541
316	106,028	KBrS015K01	AC189656	3	At4	16,738,678	16,814,214	75,536
317	136,177	KBrH004A18	AC189541	3	At4	16,808,914	16,999,511	190,597
318	292,455	KBrH009I12	AC189569	3	At4	16,982,182	17,287,050	304,868
		KBrB048F07	AC189373	3				
319	159,870	KBrH005C21	AC189549	3	At4	17,099,144	17,366,065	266,921
320	140,229	KBrB065B14	AC232512	3	At4	17,385,074	17,524,875	139,801
321	157,391	KBrB006J12	AC189204	2	At4	17,556,186	17,732,606	176,420
322	124,849	KBrH005P10	AC189552	2	At4	17,692,692	17,778,311	85,619
323	99,407	KBrB001A20	AC189183	3	At4	17,733,174	17,811,595	78,421
324	240,479	KBrB091M07	AC189521	3	At4	18,069,835	18,404,575	334,740

		KBrS004J05	AC189635	3				
325	112,105	KBrB021M22	AC189260	3	At4	18,395,029	18,477,884	82,855
326	97,963	KBrB022O03	AC189267	2	At4	18,462,239	18,569,363	107,124
327	105,566	KBrB045I17	AC189362	3	At5	144,302	367,649	223,347
328	206,564	KBrS009G14	AC189646	3	At5	396,923	722,955	326,032
		KBrB041F23	AC190052	2				
329	225,372	KBrB073I16	AC232522	3	At5	829,262	1,147,622	318,360
		KBrB092L14	AC189528	2				
330	205,452	KBrB053D06	AC189388	3	At5	1,119,742	1,630,576	510,834
		KBrS003K07	AC189631	3				
331	166,828	KBrB005N03	AC189201	3	At5	1,612,087	1,807,468	195,381
332	262,787	KBrB055N13	AC189396	2	At5	1,722,029	2,245,087	523,058
		KBrB046M04	AC189367	3				
333	130,378	KBrB004B12	AC189195	3	At5	2,241,477	2,364,229	122,752
334	102,275	KBrB001B07	AC189184	2	At5	2,356,597	2,512,524	155,927
335	140,945	KBrB038E05	AC189332	3	At5	2,505,500	2,683,586	178,086
336	138,600	KBrH006C14	AC189554	3	At5	2,709,749	2,964,973	255,224
337	130,281	KBrB034N10	AC189313	3	At5	2,838,461	3,024,564	186,103
338	225,149	KBrH052O08	AC155342	3	At5	3,031,954	3,428,129	396,175
		KBrB005M10	AC232442	2				
339	106,454	KBrH004D11	AC155341	3	At5	3,092,145	3,285,925	193,780
340	158,393	KBrB054O10	AC189393	3	At5	3,326,491	3,595,049	268,558
341	132,912	KBrB010H02	AC232450	3	At5	3,668,801	3,830,017	161,216
342	128,925	KBrB080E24	AC189473	2	At5	3,775,715	3,948,386	172,671
343	99,312	KBrB035A14	AC189315	3	At5	3,936,575	4,026,989	90,414
344	326,860	KBrH014H10	AC189599	3	At5	4,023,728	4,514,799	491,071
		KBrB054G07	AC189391	3				
		KBrB008I08	AC189212	3				
345	217,320	KBrH009B23	AC189564	3	At5	4,497,192	4,785,025	287,833
		KBrB012O13	AC189227	3				
346	114,993	KBrB013N08	AC189232	3	At5	4,784,222	4,931,962	147,740
347	266,860	KBrH003M07	AC232539	3	At5	4,926,724	5,357,944	431,220
		KBrH013I08	AC232554	3				
		KBrB018H04	AC232456	1				
		KBrB016B21	AC189239	1				
348	104,381	KBrB087B10	AC189501	3	At5	5,489,777	5,600,214	110,437
349	246,182	KBrH015C19	AC189602	2	At5	5,589,867	5,937,840	347,973
		KBrB071M18	AC189450	3				
350	156,075	KBrB015N02	AC189238	2	At5	5,873,779	6,043,796	170,017
351	157,533	KBrB069I05	AC189440	2	At5	6,041,609	6,316,955	275,346
352	121,695	KBrB004L02	AC189197	3	At5	6,261,242	6,553,121	291,879
353	145,897	KBrB039G17	AC189336	3	At5	6,482,589	6,648,858	166,269
354	60,228	KBrB001J13	AC189186	3	At5	6,684,358	6,732,668	48,310
355	158,169	KBrB067F22	AC189430	3	At5	6,689,237	6,846,437	157,200
356	178,166	KBrB060E11	AC189411	3	At5	6,845,897	7,068,585	222,688
		KBrS004M05	AC189636	2				
357	108,004	KBrH012D09	AC189583	3	At5	7,119,296	7,203,009	83,713
358	118,434	KBrB070J05	AC189442	2	At5	7,199,235	7,338,119	138,884
359	142,402	KBrB063C05	AC189418	2	At5	7,315,931	7,565,728	249,797
360	106,221	KBrB036L21	AC189323	1	At5	7,557,912	7,683,960	126,048
361	122,115	KBrB086G22	AC189497	2	At5	7,787,188	7,968,129	180,941
362	104,841	KBrB068N22	AC232516	2	At5	8,005,817	8,157,507	151,690

363	104,576	KBrB023I24	AC232463	3	At5	8,270,198	8,345,899	75,701
364	135,950	KBrB005E24	AC190048	2	At5	8,339,489	8,713,868	374,379
365	106,013	KBrB091J13	AC189520	3	At5	8,753,029	8,883,255	130,226
366	103,620	KBrB036J07	AC189321	3	At5	8,755,877	8,997,045	241,168
367	87,674	KBrH125N23	AC172883	3	At5	9,147,173	9,287,531	140,358
368	157,225	KBrB018F22	AC232455	2	At5	9,171,884	9,351,642	179,758
369	134,499	KBrB059L17	AC189409	2	At5	9,343,645	9,515,121	171,476
370	112,158	KBrS016J18	AC189657	3	At5	9,681,727	9,863,828	182,101
371	113,339	KBrH011I15	AC189579	3	At5	9,688,228	9,967,317	279,089
372	407,135	KBrB051J22	AC232494	2	At5	15,783,073	16,562,317	779,244
		KBrH005F16	AC232541	2				
		KBrH010J09	AC232548	3				
		KBrB057E04	AC232503	2				
		KBrH006F04	AC232543	3				
373	101,009	KBrH007I21	AC232546	3	At5	16,736,020	16,826,972	90,952
374	139,923	KBrB061E18	AC189413	3	At5	17,199,198	17,401,959	202,761
375	132,905	KBrH013D23	AC189591	3	At5	17,398,825	17,606,780	207,955
376	156,359	KBrB057L05	AC189402	3	At5	17,550,820	17,756,735	205,915
377	157,585	KBrB030K23	AC232475	1	At5	17,736,585	17,872,313	135,728
		KBrB053J19	AC189389	2				
378	125,002	KBrS008G07	AC189642	2	At5	17,846,252	18,030,934	184,682
379	375,125	KBrS016L08	AC189658	2	At5	18,261,716	18,863,317	601,601
		KBrH003E13	AC189537	3				
		KBrB088D23	AC189505	2				
		KBrB007O13	AC189210	3				
380	218,745	KBrB034G01	AC189310	2	At5	18,910,220	19,104,121	193,901
		KBrB017K02	AC189245	3				
381	116,940	KBrB021P11	AC189261	3	At5	19,104,131	19,226,059	121,928
382	151,038	KBrH093K03	AC155347	3	At5	19,195,358	19,362,554	167,196
		KBrH077A05	AC155343	3				
383	152,302	KBrH067N03	AC189611	2	At5	19,382,256	19,752,992	370,736
384	122,199	KBrB024J16	AC189275	3	At5	19,738,736	19,892,891	154,155
385	128,797	KBrS013H10	AC232578	3	At5	19,975,425	20,119,629	144,204
386	151,225	KBrB086B23	AC232530	3	At5	20,305,284	20,518,683	213,399
387	210,049	KBrB060J18	AC232507	3	At5	20,638,501	20,925,850	287,349
		KBrS010I13	AC232576	3				
388	124,893	KBrB003D07	AC232439	3	At5	20,974,404	21,171,633	197,229
389	200,624	KBrB059C07	AC232505	1	At5	21,219,589	21,606,965	387,376
		KBrB006G23	AC232446	3				
390	147,625	KBrB078H21	AC189470	2	At5	21,670,125	21,836,793	166,668
391	116,755	KBrB054N05	AC189392	1	At5	21,932,775	22,096,212	163,437
392	188,923	KBrB071K22	AC189448	3	At5	22,093,469	22,273,022	179,553
		KBrB001N22	AC189188	3				
393	246,844	KBrB030F12	AC189303	3	At5	22,267,965	22,620,042	352,077
		KBrH004B20	AC189542	3				
394	277,732	KBrB049H14	AC189378	3	At5	22,616,643	23,008,452	391,809
		KBrB019M05	AC189252	1				
		KBrB069F08	AC189438	3				
395	130,186	KBrB027E01	AC232472	2	At5	23,017,387	23,147,652	130,265
396	187,861	KBrH013B13	AC232552	3	At5	23,242,223	23,379,930	137,707
		KBrB081M20	AC189479	3				
397	121,155	KBrB071P24	AC189451	3	At5	23,355,243	23,574,332	219,089

398	183,104	KBrB043M07	AC189355	3	At5	23,551,865	23,735,429	183,564
		KBrB092B07	AC189523	2				
399	124,572	KBrS010D10	AC189648	3	At5	23,803,968	24,015,084	211,116
400	131,358	KBrH007A03	AC189559	2	At5	24,000,161	24,096,518	96,357
401	132,564	KBrB077C20	AC232523	2	At5	24,219,176	24,293,234	74,058
402	123,955	KBrB048M01	AC189376	2	At5	24,260,860	24,324,800	63,940
403	130,740	KBrB036M09	AC189324	3	At5	24,391,463	24,469,640	78,177
404	144,401	KBrB051M06	AC232495	3	At5	24,499,769	24,714,303	214,534
405	116,616	KBrH005L20	AC232542	3	At5	24,678,513	24,771,390	92,877
406	102,021	KBrB054D07	AC232498	2	At5	24,870,297	24,938,053	67,756
407	719,626	KBrH014E13	AC189597	3	At5	24,997,916	26,019,735	1,021,819
		KBrB036H12	AC189320	3				
		KBrB035I03	AC189316	2				
		KBrS016N10	AC189659	2				
		KBrB083J11	AC232526	3				
		KBrB007I19	AC189207	3				
		KBrH080C09	AC166741	3				
408	189,770	KBrB022L12	AC189266	3	At5	26,161,536	26,384,926	223,390
		KBrH038N24	AC232560	2				
		KBrB092J12	AC189526	3				
409	115,577	KBrB011P07	AC189225	3	At5	26,545,903	26,672,860	126,957
410	58,298	KBrB021J09	AC189258	3	At5	26,899,486	26,923,230	23,744

Table S2. Location of sequence contigs on the *B. rapa* chromosomes according to a combination of genetic map position, FISH results, physical map contig, and positional information from *A. thaliana* counterparts.

Brassica rapa									*Arabidopsis thaliana*		
Chr.	Sequence contigs	Constituent BACs	Genetic map[a]				FISH	Physical map contig[b]	Chr.	Putative counterpart[c]	
			JWF3P		VCS						
			Marker	cM	Marker	cM				Start	End
A1	318	KBrH009I12			KS40910	2.4			At4	16,982,182	17,287,050
		KBrB048F07									
	320	KBrB065B14			KS40940	2.4			At4	17,385,074	17,524,875
	321	KBrB006J12	KS40950	0.0					At4	17,556,186	17,732,606
	325	KBrB021M22	KA41000	3.5					At4	18,395,029	18,477,884
	326	KBrB022O03	KS41010	7.6					At4	18,462,239	18,569,363
	324	KBrS004J05							At4	18,069,835	18,404,575
		KBrB091M07			KA40980-3	11.1					
	323	KBrB001A20	KS40971	10.9					At4	17,733,174	17,811,595
	351	KBrB069I05			KS50350	12.7			At5	6,041,609	6,316,955
	314	KBrB092P05	KS40870	25.1					At4	16,032,221	16,577,646
		KBrB036M17	KS40860	26.9							
		KBrB052L10			KS40850	15.1					
		KBrS004O21	KS40840	28.5	KS40840	17.5					
	312	KBrB066A08	KS40810	34.0	KS40810	21.3			At4	15,490,841	15,794,981
		KBrB010F19	KS40800	37.2							
		KBrB035P03									
	310	KBrB010F06	KS40780	39.5					At4	15,127,469	15,384,533
		KBrH011G12	KR40770-1	40.5							
	308	KBrB080E18	KR40750-2	42.9					At4	14,825,787	14,963,233
	303	KBrS003F17			KS40690	30.6			At4	14,053,272	14,158,065
	284	KBrB021L13	KC40420-3	53.0					At4	10,897,677	11,018,095
	276	KBrS005I11			KBrS005I11-5	30.7			At4	9,778,327	9,889,268
	280	KBrB023N19							At4	10,283,277	10,500,757
		KBrB060L22	KS40380	56.2							
	285	KBrB085D18	KA40440-2	58.2	KA40440-2	36.8			At4	11,394,747	11,517,701
	286	KBrB084N21	KS40450	61.6					At4	11,529,699	11,546,705

	287	KBrB055A02						At4	11,580,286	11,725,379
		KBrB069A15	KA40470-3	63.6	KA40470-3	37.3				
	289	KBrB001M05	KA40500-2	64.6				At4	12,053,350	12,173,730
	292	KBrH015O11						At4	12,392,256	12,533,978
		KBrB042G14			KA40550-1	42.9				
	293	KBrH001M22	KC40560-1	70.3				At4	12,532,826	12,626,260
	296	KBrB061L05	KC40590-4	71.7	KA40590-4	44.7		At4	12,904,019	12,986,316
	343	KBrB035A14	KS50200	74.3	KS50200	51.1		At5	3,936,575	4,026,989
	301	KBrH009D02	KS40660	78.3	KS40660	47.9		At4	13,904,104	13,941,737
	209	KBrB048K11	KR30650-1	81.9				At3	8,679,184	8,732,355
	202	KBrB042J11	KS30540	83.9				At3	7,399,178	7,475,302
	273	KBrH122A22	KS40282	87.6				At4	9,580,351	9,721,375
	188	KBrB089M05	KS30360	93.2				At3	5,296,316	5,398,899
	177	KBrH003N18	KS30210	119.0				At3	3,007,564	3,224,660
	170	KBrB078A03	KBrB078A03-2	154.7				At3	1,119,931	1,256,622
	167	KBrB020F06					FISH	At3	363,958	466,087
A2	376	KBrB057L05	KR50650-3	20.5				At5	17,550,820	17,756,735
	328	KBrS009G14			KS50020	0.0		At5	396,923	722,955
		KBrB041F23								
	330	KBrB053D06			KA50060	0.1		At5	1,119,742	1,630,576
		KBrS003K07								
	339	KBrH004D11			KS50164	0.1		At5	3,092,145	3,285,925
	340	KBrB054O10	KS50170	26.7				At5	3,326,491	3,595,049
	346	KBrB013N08	KS50260	36.6				At5	4,784,222	4,931,962
	349	KBrH015C19						At5	5,589,867	5,937,840
		KBrB071M18	KS50330	41.2	KS50330	16.2				
	356	KBrB060E11						At5	6,845,897	7,068,585
		KBrS004M05	KS50410	43.8	KS50410	22.6				
	358	KBrB070J05			KS50430	25.5		At5	7,199,235	7,338,119
	402	KBrB048M01			KS51030	29.3		At5	24,260,860	24,324,800
	400	KBrH007A03	KC51020-4	46.4	KS51020	33.9		At5	24,000,161	24,096,518
	394	KBrB069F08	KS50940	50.4				At5	22,616,643	23,008,452
		KBrB019M05								
		KBrB049H14	KS50920	50.7						

	390	KBrB078H21	KS50860	52.8	KS50860	38.4		At5	21,670,125	21,836,793
	392	KBrB071K22						At5	22,093,469	22,273,022
		KBrB001N22	KS50890	54.1						
	387	KBrB060J18	KBrB060J18-5	56.0				At5	20,638,501	20,925,850
		KBrS010I13								
	076	KBrB070J11			KA11120-2	40.8		At1	24,178,632	24,339,139
	078	KBrB010O09	KS11140	62.2				At1	24,624,596	24,752,376
	080	KBrB005J17			KS11160	42.0		At1	25,086,237	25,218,162
	089	KBrB084M08	KR11300-1	64.0				At1	26,601,905	26,710,275
	088	KBrB034I14	KR11290-1	64.5				At1	26,485,125	26,603,147
	136	KBrB052E10	KS20580d	68.6				At2	14,074,943	14,131,503
	235	KBrB084F02	KS31020	71.9				At3	20,487,724	20,616,681
	222	KBrH014A01			KBrH014A01-5	54.1		At3	11,047,187	11,295,128
	220	KBrB027J02			KA20090	57.6		At3	10,789,218	11,076,897
	261	KBrB021M05	KA40120-2	77.1				At4	2,325,516	2,433,742
	264	KBrB007I08	KBrB007I08-4	83.8				At4	6,609,359	6,804,395
	385	KBrS013H10			KA50790-2	64.7		At5	19,975,425	20,119,629
	371	KBrH011I15			KS50540	67.2		At5	9,688,228	9,967,317
	369	KBrB059L17			KA50530-1	68.7		At5	9,343,645	9,515,121
	365	KBrB091J13			KS50500	72.8		At5	8,753,029	8,883,255
	361	KBrB086G22			KS50460	76.6		At5	7,787,188	7,968,129
	375	KBrH013D23			KA50640	81.0		At5	17,398,825	17,606,780
	407	KBrH080C09	KS51151_MAF1	96.8				At5	24,997,916	26,019,735
		KBrB007I19								
		KBrB083J11								
		KBrS016N10			KS51120	83.0				
		KBrB035I03								
		KBrB036H12			KS51100	83.4				
		KBrH014E13			KS51090	84.0				
	405	KBrH005L20			KA51081	86.3		At5	24,678,513	24,771,390
	410	KBrB021J09	KS51180	102.3				At5	26,899,486	26,923,230
A3	322	KBrH005P10					FISH	At4	17,692,692	17,778,311
	315	KBrB010M19	KS40880	25.1				At4	16,567,755	16,740,296
	313	KBrH009C15						At4	15,794,504	16,147,244

	KBrB043B23	KR40830-2a	26.1	KA40830-3	3.1					
	KBrH029A20									
311	KBrB079E08			KBrB079E08	4.0			At4	15,419,858	15,590,608
305	KBrH015E17					FISH		At4	14,360,811	14,572,070
	KBrB070M16									
302	KBrB069M23			KS40670	5.4			At4	13,936,422	14,084,157
	KBrB021P17			KS40680	5.8					
300	KBrB014O06			KS40650	7.3			At4	13,735,685	13,853,801
298	KBrB045B23							At4	13,131,259	13,345,983
	KBrB028P01	KBrB028P01-4	27.5							
294	KBrH005A08	KBrH005A08-2	37.0					At4	12,591,032	12,701,134
291	KBrB091M11							At4	12,278,902	12,429,912
	KBrB053E08			KBrB053E08	12.7					
290	KBrS011B08			KS40510	13.6			At4	12,177,875	12,280,349
282	KBrB085J21	KS40400	38.7					At4	10,598,343	10,817,097
223	KBrS011C11	KS30810	39.3					At3	17,525,154	17,709,961
279	KBrH006A08			KS40350	17.1			At4	10,055,311	10,314,578
	KBrB004G11									
275	KBrH045E23	KR40313	41.0					At4	9,726,100	9,876,760
271	KBrB047I09							At4	8,965,126	9,432,371
	KBrB026A07									
	KBrS003O10			KS40270	17.1					
	KBrB084K23									
270	KBrB089H07	KS40250	42.8	KS40250	20.1			At4	8,696,630	8,909,662
226	KBrB013J16							At3	18,430,289	18,616,070
	KBrB043L22	KC30870-3	45.6							
228	KBrB002E24	KC30890-1	46.2					At3	18,866,027	18,929,517
230	KBrB084F01			KS30930	21.5			At3	18,956,714	19,477,537
	KBrS012D09	KS30920	47.6	KS30920	24.3					
	KBrS001M03			KS30910	24.5					
111	KBrB058B22							At2	1,581,646	2,183,883
	KBrS008C11	KS20080	53.8							
	KBrB011D06									
	KBrB037E22									

112	KBrH001K17			KA20130	31.9		At2	6,315,474	6,519,481
	KBrB091E13	KA20140	55.8						
211	KBrB072K21	B072K21-5	58.3				At3	8,999,019	9,155,094
210	KBrB084M06			KS30660	37.7		At3	8,726,428	8,906,040
208	KBrB026F22					FISH	At3	8,140,042	8,708,225
	KBrB073E20								
206	KBrB037A01	KC30610-2	62.7				At3	7,914,810	8,108,334
204	KBrB059A03					FISH	At3	7,604,138	7,818,240
201	KBrB009B09			KS30530	44.3		At3	7,189,135	7,451,659
198	KBrB044L16	KS30500	64.1				At3	6,840,937	7,061,195
192	KBrB019B16	KS30430	66.1				At3	6,031,185	6,428,658
	KBrB070K15								
189	KBrB010F13						At3	5,371,430	5,796,685
	KBrB052N08			KS30380	45.0				
	KBrB036B21								
186	KBrB021F10	KS30350	69.5				At3	5,090,380	5,273,179
184	KBrB047D06	KS30300	73.0				At3	4,356,247	5,037,685
	KBrH001D20								
	KBrB019N06								
	KBrB042O05			KS30330	46.3				
181	KBrH122D17			KS30251	47.3		At3	3,633,800	3,871,449
176	KBrB055G10	KS30200	75.8	KS30200	49.9		At3	2,823,519	3,007,826
173	KBrB003A10						At3	1,618,909	2,242,276
	KBrH013F17	KBrH013F17-3	78.1						
	KBrB013O20								
	KBrH005H18								
172	KBrH012A23	KR30090-2	79.5				At3	1,488,871	1,659,090
171	KBrB043L02						At3	1,259,233	1,513,414
	KBrB037C07			KS30080	52.6				
169	KBrS007P17			KC30030	53.8		At3	399,330	1,196,133
	KBrB001H24	KS30040	80.2	KS30040	54.6				
	KBrB058F21								
166	KBrB049D17	KBrB049D17-2	83.3	KBrB049D17-4	55.7		At3	28,394	337,524
	KBrB055E21								

174	KBrH011G10	KS30140	84.1					At3	2,241,452	2,438,892
257	KBrB027P21							At4	1,064,350	1,241,141
	KBrH129I15			H129I15-2	61.9					
265	KBrH009K01			KS40170	64.5			At4	6,950,272	7,213,014
262	KBrB061C02			KA40130	65.9			At4	5,785,078	6,176,960
386	KBrB086B23			KS50800	66.7			At5	20,305,284	20,518,683
263	KBrB051E01			KA40140	67.9			At4	6,270,734	6,657,116
128	KBrB038M13	KS20420	90.0					At2	11,436,264	11,762,408
132	KBrB068E07	KS20470	92.4					At2	12,216,771	12,627,902
159	KBrB056I08	KR20910-3	93.7					At2	18,321,704	18,625,954
154	KBrB086M08			KBrB086M08-2	72.8			At2	17,156,790	17,316,783
150	KBrB032C14					FISH		At2	16,536,181	16,706,914
144	KBrH007P05	KR20690-2b	104.4					At2	15,506,991	15,642,630
142	KBrB031G07			KC20670-3	81.2			At2	15,183,632	15,329,601
139	KBrS012M03	KS20620	109.0					At2	14,563,971	14,938,374
	KBrB042N23									
056	KBrB065E07			KS10870	88.3			At1	12,355,953	12,655,972
134	KBrB080O07							At2	12,802,153	14,060,783
	KBrB082C18									
	KBrH003D18			KA20550	89.1					
	KBrB074K06									
	KBrB068H20			KC20530-3	90.6					
	KBrH005J02									
	KBrB071A06									
	KBrB026F03									
	KBrH038M21	KR20491_FLC5	110.3							
130	KBrB019J14			KC20450-2	90.6			At2	11,964,165	12,020,610
388	KBrB003D07	KC50830-2	115.6				1278	At5	20,974,404	21,171,633
389	KBrB059C07						1278	At5	21,219,589	21,606,965
	KBrB006G23									
391	KBrB054N05	KS50870	118.4					At5	21,932,775	22,096,212
393	KBrB030F12			KS50900	91.5			At5	22,267,965	22,620,042
	KBrH004B20									
395	KBrB027E01			KBrB027E01	94.0			At5	23,017,387	23,147,652

	397	KBrB071P24	KS50980	120.3	KS50980	94.1		At5	23,355,243	23,574,332
	133	KBrS012H21			KBrS012H21-3	96.1		At2	12,675,449	12,777,366
	163	KBrH011O17	KS11150	126.1				At2	18,824,314	19,409,344
		KBrB050C17								
		KBrB049N04								
	352	KBrB004L02	KS50360	126.6				At5	6,261,242	6,553,121
	347	KBrH003M07						At5	4,926,724	5,357,944
		KBrH013I08	KC50280-1	128.2	KS50280	107.4				
		KBrB018H04	KR50290-2	129.4						
		KBrB016B21			KS50300	107.4				
	252	KBrS008K03			KS31210	109.8		At3	23,246,113	23,329,630
	344	KBrH014H10	KS50210	134.6				At5	4,023,728	4,514,799
		KBrB054G07			KA50220	111.1				
		KBrB008I08								
	341	KBrB010H02	KBrB010H02	137.1				At5	3,668,801	3,830,017
	338	KBrH052O08	KR50161_FLC3	146.6				At5	3,031,954	3,428,129
		KBrB005M10								
	336	KBrH006C14	KS50140	148.9	KS50140	114.4		At5	2,709,749	2,964,973
	334	KBrB001B07			KA50120-1	115.2		At5	2,356,597	2,512,524
	332	KBrB055N13	KS50090	151.1				At5	1,722,029	2,245,087
		KBrB046M04								
	327	KBrB045I17	KR50010	153.2				At5	144,302	367,649
	329	KBrB073I16						At5	829,262	1,147,622
		KBrB092L14	KS50050	160.5						
A4	231	KBrB063M21					FISH	At3	19,477,022	19,581,652
	236	KBrB056C05	KS31030	27.2				At3	20,616,574	20,693,445
	127	KBrH010F07	KS20410	41.3	KS20410	12.3		At2	11,056,282	11,544,701
		KBrB060F02	KR20400-1	41.8	KS20400	12.3				
	129	KBrB014N06			KS20430	13.5		At2	11,747,884	11,968,358
		KBrS006L21								
	131	KBrB028K05			KS20460	17.3		At2	12,133,929	12,230,279
	135	KBrB084C18			KA20560-2	26.8		At2	13,813,476	13,949,978
	137	KBrB026E08	KS20600	54.8	KS20600	27.0		At2	14,084,258	14,293,678
		KBrS011C02	KS20590	60.3						

	140	KBrB089M13	KS20640	62.9	KS20640	28.7		At2	14,922,715	15,061,692
	055	KBrB041L12			KBrB041L12-2	34.4		At1	12,127,931	12,217,449
	143	KBrS003G14	KA20680-2	64.2				At2	15,326,352	15,534,179
	147	KBrB089D07	KS20730	67.6				At2	15,769,349	15,952,731
		KBrB033O04	KS20720	68.5						
	148	KBrH004D08	KS20740	70.5				At2	15,969,610	16,254,366
		KBrB002P20	KR20750-1	75.2						
	151	KBrS005G02			KA20790	43.4		At2	16,679,924	16,825,168
	153	KBrH012A14			KBrH012A14_1	50.9		At2	16,913,963	17,160,444
	155	KBrH009I04	KS20840	78.7				At2	17,258,946	17,498,961
	152	KBrH014A02	KR20810-4	80.0	KC20810-4	51.1		At2	16,810,135	16,944,672
		KBrB092D10	KR20800-1	83.0						
	162	KBrB073H13	KBrB073H13-2	87.9				At2	18,731,468	18,926,036
A5	165	KBrB056F17			KBrB056F17-2	0.7		At3	8,724	74,005
	168	KBrB024M19			KBrB024M19	2.2		At3	363,958	441,040
	175	KBrB042L19	KA30150-2	15.2				At3	2,388,020	2,671,655
		KBrB072L17	KS30160	17.9	KS30160	16.7				
		KBrB047A09								
	180	KBrH004P05	KS30240	28.6	KS30240	21.7		At3	3,557,024	3,689,492
	178	KBrB087P06	KS30220	30.1	KS30220	21.8		At3	3,206,586	3,360,332
	182	KBrB063G23	KS30260	36.1	KA30260-3	22.7		At3	3,860,353	4,300,419
		KBrB039A19	KS30270	40.4	KS30270	24.6				
		KBrB069G15			KA30280	25.8				
	183	KBrB053K16			KA30290	27.8		At3	4,209,634	4,368,149
	185	KBrH001C24	KS30340	48.1				At3	5,027,750	5,133,999
	190	KBrB042O03					FISH	At3	5,775,290	5,892,325
	191	KBrH013M23	KA30410	58.8				At3	5,845,360	5,940,753
	193	KBrB071H12	KS30450	62.7				At3	6,434,763	6,508,753
	195	KBrB077L14			KA30470	34.7		At3	6,632,413	6,683,192
	196	KBrB082I19			KS30480	36.3		At3	6,681,883	6,785,062
	197	KBrB017O23			KBrB017O23-1	37.2		At3	6,782,832	6,837,517
	200	KBrB045I12			KS30520	41.1		At3	7,089,206	7,189,368
	203	KBrB092B03						At3	7,475,296	7,627,760
		KBrB046C11	KA30560-1	72.7						

	207	KBrB072L19					FISH	At3	8,113,223	8,161,031
	052	KBrB026E21			KBrB026E21-S1	42.4		At1	11,394,679	11,470,983
	058	KBrH080L24					FISH	At1	17,700,798	17,720,627
	060	KBrH012G24			KS10940	42.6		At1	18,105,624	18,266,210
	067	KBrH001D10	KS11020	70.6				At1	20,712,120	20,883,081
	243	KBrB010L05			KS31110	42.6		At3	21,565,025	21,750,864
	205	KBrS005O12						At3	7,835,162	7,956,875
		KBrB039P21			KA30600	42.9				
	269	KBrB085G17	KC40240-3	81.1				At4	8,611,816	8,715,243
	399	KBrS010D10			KA51010	59.1		At5	23,803,968	24,015,084
	141	KBrB050K06	KS20650	86.7				At2	15,010,714	15,218,310
		KBrS010A05								
	145	KBrH015L23	KS20700	89.7				At2	15,588,469	15,694,430
	146	KBrB082L07	KS20710	92.4				At2	15,689,120	15,773,066
	149	KBrB023F24	KS20760	101.6	KS20760	67.4		At2	16,222,045	16,565,145
		KBrB051I12								
	157	KBrB014C24					FISH	At2	17,849,042	17,954,664
	160	KBrB016N13					FISH	At2	18,651,165	18,694,950
	164	KBrB061N03			KS20960	69.7		At2	19,400,748	19,688,436
		KBrB042E01								
		KBrS004I08	KR20980-1	102.9						
	161	KBrB086L12	KR20930-1	110.7				At2	18,685,009	18,809,342
	158	KBrH014M21	KS20890	122.9				At2	17,947,962	18,102,484
	156	KBrH117N09	KS20851	132.1	KS20851	79.2		At2	17,476,767	17,765,476
		KBrB010H06	KS20860	134.0						
		KBrH012N11								
	283	KBrB067M19	KS40410	156.0				At4	10,806,216	10,951,575
A6	065	KBrH003K23			KS11000a	2.9		At1	20,189,187	20,587,238
	064	KBrS012H18	KR10990-1	0.0	KS10990	3.2		At1	19,525,342	19,795,407
		KBrB022P06	KS10980	3.9	KS10980	3.2				
	063	KBrB018N18	KS10970	7.9	KS10970	4.0		At1	19,194,382	19,415,194
	062	KBrB030D08			KS10960	6.2		At1	18,665,629	18,865,779
		KBrH109M07								
	061	KBrB065N20	KS10950	11.7	KS10950	7.2		At1	18,514,338	18,660,591

016	KBrB086J10	KS10190	31.7					At1	3,483,323	3,608,103
020	KBrB086M23	KS10280	43.1	KS10280	31.1		123	At1	4,955,927	5,077,020
022	KBrB056G18						123	At1	5,132,667	5,236,043
023	KBrB034J13	KC10310-2	54.0					At1	5,231,475	5,306,884
025	KBrB041H16							At1	5,687,229	5,839,801
	KBrB049E19	KA10330-1	59.4							
030	KBrS007I12							At1	6,628,911	6,859,002
	KBrB043O20	KS10400	70.7							
	KBrH006P24			KS10410	34.5					
032	KBrB018K09							At1	7,150,534	7,387,905
	KBrB011L09	KS10460	71.7							
	KBrB076B03			KS10470	34.8					
213	KBrH010N11			KA30690	36.0			At3	9,460,772	9,713,516
	KBrB007A19									
224	KBrB044D19			KS30830	37.3			At3	18,159,673	18,328,481
	KBrB062J16	KS30840	73.6							
225	KBrB051E13			KBrB051E13-7	44.1			At3	18,317,038	18,422,496
115	KBrB023O12			KBrB023O12-1	46.5			At2	7,571,972	7,658,534
116	KBrH004A02			KBrH004A02-1	51.0			At2	7,700,726	7,845,353
	KBrB024N11									
363	KBrB023I24	KS50480	90.2					At5	8,270,198	8,345,899
368	KBrB018F22	KBrB018F22	92.8					At5	9,171,884	9,351,642
370	KBrS016J18	KS50550	95.5					At5	9,681,727	9,863,828
221	KBrH013N10	KS30770	105.5					At3	10,941,793	11,137,871
219	KBrH006L19			KS30761	70.4			At3	10,502,551	10,850,227
218	KBrS004A14			KS30740	76.1			At3	10,252,668	10,446,543
216	KBrB004L01					FISH		At3	9,822,560	10,029,091
215	KBrB089H11	KS30720	109.5					At3	9,752,271	9,939,295
109	KBrH009H15	KS20050	114.2					At2	867,367	1,031,444
194	KBrH054E04			KA30461	86.6			At3	6,496,206	6,599,020
212	KBrB008D15			KS30680	89.3			At3	9,303,078	9,388,602
106	KBrB051H12			KS20010	93.0			At2	170,829	285,026
381	KBrB021P11	KS50760	125.8					At5	19,104,131	19,226,059
380	KBrB034G01	KS50740	128.4	KS50740	101.0			At5	18,910,220	19,104,121

		KBrB017K02									
	379	KBrB007O13	KS50720	130.6					At5	18,261,716	18,863,317
		KBrB088D23									
		KBrH003E13	KS50700	133.7							
		KBrS016L08	KR50690-2	138.1							
	378	KBrS008G07	KS50670	142.6					At5	17,846,252	18,030,934
	374	KBrB061E18	KS50630	144.7					At5	17,199,198	17,401,959
A7	120	KBrH001J06	KS20240	20.8	KS20240	0.8			At2	8,729,574	8,777,026
	118	KBrB022C05	KS20230	24.7					At2	8,467,325	8,513,989
	048	KBrS003D10	KS10730	30.1	KS10730	4.7			At1	10,779,364	10,896,101
	047	KBrB042D24			KBrB042D24-3	6.5			At1	10,728,983	10,819,265
	038	KBrB080N15	KS10570	39.6					At1	8,853,999	8,998,123
	033	KBrB019O05			KBrB019O05-5	10.6			At1	7,400,068	7,607,484
	372	KBrB051J22							At5	15,783,073	16,562,317
		KBrH005F16			KC50590-1	11.8					
		KBrH010J09			KS50600	22.3					
		KBrB057E04									
		KBrH006F04									
	373	KBrH007I21	KA50620-1	47.9					At5	16,736,020	16,826,972
	233	KBrB023N04							At3	19,858,026	20,313,959
		KBrB048D12			KA30990-1	24.1					
	237	KBrB084H08			KS31040	25.5			At3	20,702,640	20,954,147
	242	KBrB070L01	KS31100	64.4				140	At3	21,453,642	21,605,399
	244	KBrS009G24			KS31120	28.5		140	At3	21,746,995	21,944,467
	246	KBrB027O09	KR31140-5	64.7	KS31140	31.6		140	At3	22,021,638	22,349,030
	248	KBrB025O08						140	At3	22,484,734	22,784,532
	250	KBrH007M24			KS31190	32.7			At3	22,789,915	22,892,689
	105	KBrB026K21							At1	30,024,834	30,406,480
		KBrB044D01			KS11550	38.5					
		KBrB034F07			KS11560	39.4					
	101	KBrB026G01							At1	28,377,740	29,072,827
		KBrB041J04									
		KBrB006H21	KR11460-3a	53.6	KS11460	45.3					
	100	KBrB084P16					FISH		At1	28,197,092	28,409,157

		KBrH012I05								
	098	KBrH015N11			KS11400	50.1		At1	27,990,760	28,149,736
	095	KBrH014G16			KBrH014G16-1	52.9		At1	27,579,556	27,789,405
	094	KBrB086N06	KS11360	56.3				At1	27,450,228	27,610,965
	232	KBrB087M20	KA30950	58.9				At3	19,636,956	19,817,563
		KBrB023B16								
	099	KBrB074J14	MK014	61.9				At1	28,103,624	28,205,246
	096	KBrB056L15	KS11380	63.0				At1	27,776,419	27,896,101
	097	KBrH011B08	KS11390	67.0				At1	27,875,667	28,013,097
	093	KBrB088E11					FISH	At1	27,342,093	27,450,580
	092	KBrB038O16						At1	27,025,239	27,354,025
		KBrH011C10	KR11340-1a	77.5						
	091	KBrB055K07					FISH	At1	26,907,890	27,026,563
	090	KBrB057E05			KS11310	54.5		At1	26,625,149	26,915,527
	084	KBrB026C23					FISH	At1	25,760,301	25,941,449
	083	KBrB034A02	KR11210-2a	80.0				At1	25,717,571	25,833,399
	082	KBrB084K02	KR11200-1	84.7				At1	25,377,809	25,688,010
		KBrB008G18	KBrB008G18	89.8						
	081	KBrH010F15			KS11170	59.3		At1	25,209,324	25,371,765
	079	KBrB027F23			KBrB027F23	73.3		At1	24,919,763	25,195,085
	077	KBrB083K19	KS11130	91.3				At1	24,336,204	24,556,589
	087	KBrB073K15	KS11280	93.2				At1	26,334,322	26,474,804
	085	KBrB073F16						At1	25,921,972	26,220,301
		KBrB028I01								
		KBrH012E04	KS11250	100.4						
		KBrB043L15								
	102	KBrB068K17						At1	29,017,585	29,469,444
		KBrB061K11			KS11490	93.3				
		KBrB047M06			KS11480	94.3				
		KBrB021P15								
	104	KBrB007M04			KBrB007M04-3	99.2		At1	29,753,538	30,000,682
		KBrB034L08								
A8	011	KBrB090M17	KR10130-1	0.0				At1	2,421,409	2,620,403
	012	KBrH001D23	KR10140-2	11.1				At1	2,578,342	3,073,053

	KBrH006H02								
013	KBrH013B19	KS10150	20.9				At1	2,818,630	3,009,625
014	KBrB037O12			KS10160	12.6		At1	2,994,364	3,337,521
	KBrB091G04			KS10170	15.3				
017	KBrB006B05	KS10240	24.9				At1	4,208,805	4,754,593
	KBrB017B11	KS10250	29.3	KS10250	32.3				
049	KBrB053L06	KA10740	31.5				At1	10,936,911	11,088,550
	KBrB012O24								
024	KBrB077F22	KS10320	32.4	KS10320	36.9		At1	5,300,862	5,719,276
026	KBrS004B22			KBrS004B22-2	38.4		At1	5,795,958	6,032,436
031	KBrB058M10						At1	6,883,693	7,158,620
	KBrB042N05	KS10440	35.4	KS10440	44.2				
043	KBrH006E24						At1	9,540,160	9,789,975
	KBrB003O07			KBrB003O07-6	48.4				
040	KBrB052E19	KR10610-1a	37.5	KA10610	49.2		At1	9,274,520	9,422,091
037	KBrB006C05	KS10550	41.7	KS10550	49.8		At1	8,216,008	8,434,924
034	KBrB023N08						At1	7,595,068	7,891,345
	KBrB023L08			KBrB023L08-4	51.0				
029	KBrB044C04	KA10380-2	49.0				At1	6,484,158	6,668,815
044	KBrB025C06					157	At1	9,911,938	10,031,090
045	KBrB016E20	KC10680-1	56.9	KA10680-2	54.4	157	At1	10,035,138	10,553,458
	KBrH012O06								
	KBrB046G20	KBrB046G20-1	60.7						
046	KBrB027K16	KS10710	61.4			157	At1	10,655,250	10,674,666
268	KBrB074G23	KBrB074G23-4	64.7				At4	8,500,108	8,555,202
319	KBrH005C21	KS40930	66.8				At4	17,099,144	17,366,065
317	KBrH004A18			KS40900	58.5		At4	16,808,914	16,999,511
299	KBrH004I05						At4	13,308,723	13,777,829
	KBrH012K23			KS40630	58.5	257			
297	KBrB034C07					257	At4	12,986,346	13,151,133
295	KBrB059G16	KC40580-4	68.9				At4	12,676,966	12,927,749
066	KBrH013K13	KS11010	71.4				At1	20,599,154	20,722,124
307	KBrB090J11	KS40740	74.3				At4	14,672,812	14,830,328
309	KBrB048L11	KR40760-3	75.9				At4	14,952,719	15,134,553

	304	KBrB006O19	KC40700-2	76.5					At4	14,131,340	14,345,007
	288	KBrB075L03	KS40480	78.6					At4	11,709,673	12,049,125
		KBrB006A15	KS40490	79.5							
	316	KBrS015K01	KR40890-1	80.4					At4	16,738,678	16,814,214
	272	KBrB056G23	KS40300	81.9					At4	9,452,822	9,636,949
	274	KBrH138O03	KS40314	82.9					At4	9,681,508	9,760,826
	278	KBrS008P19	KS40340	84.7					At4	9,939,736	10,063,420
	253	KBrB071M14					FISH		At3	23,328,892	23,405,716
		KBrB065N19									
A9	001	KBrB063A04	KBrB063A04-2	16.0	KBrB063A04-4	0.0			At1	31,237	166,379
	003	KBrB092L06	KS10020	23.3					At1	448,546	1,514,046
		KBrB005I07									
		KBrB090K20									
		KBrB089B13	KS10040	24.5							
		KBrB043F18	KS10050	28.8	KS10050	6.7					
		KBrB002B23									
		KBrB017P15									
	008	KBrB012F17						168	At1	2,085,721	2,281,627
	009	KBrH138P04	KS10121	38.9				168	At1	2,125,327	2,302,896
	010	KBrH034K06					FISH		At1	2,330,528	2,547,586
	015	KBrS004C14			KA10180	13.0			At1	3,281,652	3,473,638
	019	KBrH010P01			KS10270	18.5			At1	4,739,699	4,960,978
		KBrB070M24			KS10260	18.6		170			
	021	KBrB090H23						170	At1	5,064,678	5,164,600
	028	KBrH092O19			KBrH092O19	24.4			At1	6,187,585	6,424,882
	119	KBrH009P18					FISH		At2	8,505,583	8,748,385
	121	KBrH095F22							At2	8,747,195	9,518,967
		KBrB017F11									
		KBrB082F21	KC20280-2	67.4							
		KBrH081N08	KS20291	75.8							
		KBrB019I24	KS20300	77.8							
		KBrB025M01	KS20310	83.1	KS20310	29.5					
		KBrS009B06			S009B06-4	30.4					
		KBrB035J16									

	KBrH070I10								
123	KBrB043N10			KA20340	35.9		At2	9,852,483	9,996,242
126	KBrS008H22	KR20380-1	84.3				At2	10,683,946	11,113,729
	KBrB063K02								
251	KBrH110N19	KA30981	87.6				At3	22,997,995	23,193,178
249	KBrB044E18	KS31180	92.1				At3	22,732,496	22,915,902
	KBrH012J09			KBrH012J09	41.1				
247	KBrB021E01						At3	22,343,021	22,514,217
	KBrB036H01	KS31150	92.2			183			
245	KBrB081E09					183	At3	21,896,420	22,132,746
	KBrB070F08								
241	KBrB078G17			KBrB078G17-4	49.1		At3	21,343,627	21,469,580
240	KBrB013M01			KS31070	50.2		At3	21,153,305	21,353,061
	KBrB073E19								
238	KBrB073D09			KS31050	52.9		At3	20,937,927	21,062,348
234	KBrH012N08						At3	20,311,130	20,490,523
	KBrH117O12	KS31002	99.9	KS31002	57.3				
	KBrS001F19								
229	KBrB088I08			KS30900	63.8		At3	18,929,416	19,000,745
227	KBrB049N17	KS30880	104.8	KS30880	63.8		At3	18,677,362	18,775,944
035	KBrH003E21			KBrH003E21-1	64.6		At1	7,847,446	7,929,911
036	KBrH020D15						At1	8,047,504	8,250,790
	KBrB048C04	KR10540	106.2						
039	KBrB059J21	KS10600	110.9				At1	8,995,173	9,304,219
	KBrB066M16	KC10590-1	111.6	KS10590	65.3				
	KBrB089L03								
041	KBrB024J13			B024J13-2	65.9		At1	9,375,059	9,426,640
042	KBrS010I09	KS10630	113.3				At1	9,493,340	9,577,271
050	KBrH089C20						At1	11,046,912	11,222,416
	KBrB016K20	KS10760	115.6						
	KBrB070J23								
051	KBrB074J19			KS10790	72.1		At1	11,254,228	11,276,095
108	KBrB072E02	KS20030	117.5				At2	556,557	959,108
	KBrH006N19								

	110	KBrB010H08	KS20060	121.2				At2	1,149,337	1,286,020
	382	KBrH093K03						At5	19,195,358	19,362,554
		KBrH077A05	KS50774	122.4						
	075	KBrB022A09					FISH	At1	23,689,065	23,827,162
	071	KBrB092B15					FISH	At1	22,561,170	22,704,766
	070	KBrB036K20			KBrB036K20-2	73.2		At1	21,971,630	22,131,759
	069	KBrB037F09	KS11050	123.4	KS11050	73.3		At1	21,493,016	21,504,680
	068	KBrB086C10	KS11040	124.6				At1	21,132,535	21,262,720
	377	KBrB030K23						At5	17,736,585	17,872,313
		KBrB053J19	KS50660	126.1						
	073	KBrB025K04	KS11100	130.9				At1	23,529,980	23,653,002
	072	KBrB069A23	KS11090	136.8	KS11090	76.6		At1	23,375,273	23,524,264
	122	KBrH014M07	KS20260	139.3				At2	8,751,484	8,845,860
	117	KBrB059K16	KA20200	143.5				At2	7,804,284	7,988,188
		KBrH011J16								
	114	KBrH006K14			KBrH006K14-3	82.3		At2	7,217,033	7,497,459
	409	KBrB011P07	KS51170	151.0				At5	26,545,903	26,672,860
	408	KBrB022L12	KS51160	153.3				At5	26,161,536	26,384,926
		KBrH038N24								
		KBrB092J12	KS51190	156.7						
	404	KBrB051M06	KS51050	165.9				At5	24,499,769	24,714,303
	362	KBrB068N22			KS50470	94.1		At5	8,005,817	8,157,507
	364	KBrB005E24			KS50490	95.0		At5	8,339,489	8,713,868
	366	KBrB036J07			KS50510	97.3		At5	8,755,877	8,997,045
	367	KBrH125N23	KS50524	171.0				At5	9,147,173	9,287,531
	383	KBrH067N03	KS50775	175.1				At5	19,382,256	19,752,992
	217	KBrH102C07						At3	9,953,930	10,687,423
		KBrH123C19	H123C19-1	179.7						
	260	KBrB037E04	KR40090-2	188.1				At4	1,497,492	1,607,711
	258	KBrB018D16					FISH	At4	1,108,500	1,308,779
	256	KBrH121P05	KBrH121P05-2	188.1				At4	741,285	927,851
	255	KBrB037A09			KS40030	119.4		At4	447,118	536,508
	254	KBrB041F06	KA40010	198.0				At4	151,566	326,890
A10	331	KBrB005N03					FISH	At5	1,612,087	1,807,468

	333	KBrB004B12			KS50110	0.0		At5	2,241,477	2,364,229
	335	KBrB038E05					233	At5	2,505,500	2,683,586
	337	KBrB034N10	KS50150	11.3			233	At5	2,838,461	3,024,564
	342	KBrB080E24	KS50190	22.8				At5	3,775,715	3,948,386
	345	KBrB012O13			KS50250	16.3		At5	4,497,192	4,785,025
		KBrH009B23	KS50240	25.8	KS50240	18.2				
	348	KBrB087B10	KS50310	35.2				At5	5,489,777	5,600,214
	350	KBrB015N02	KS50340	39.4	KS50340	28.8		At5	5,873,779	6,043,796
	353	KBrB039G17			KS50370	29.6		At5	6,482,589	6,648,858
	354	KBrB001J13	KR50380-3a	42.1	KS50380	30.2		At5	6,684,358	6,732,668
	359	KBrB063C05	KR50440-1a	44.4				At5	7,315,931	7,565,728
	360	KBrB036L21	KS50450	46.0	KS50450	32.1		At5	7,557,912	7,683,960
	355	KBrB067F22	KS50390	47.1				At5	6,689,237	6,846,437
	103	KBrB006F18	KS11510	47.7				At1	29,467,089	29,801,678
	357	KBrH012D09	KS50420	49.5				At5	7,119,296	7,203,009
	403	KBrB036M09			KS51040	32.1	241	At5	24,391,463	24,469,640
	401	KBrB077C20					241	At5	24,219,176	24,293,234
	398	KBrB043M07	KS50990	52.0				At5	23,551,865	23,735,429
		KBrB092B07			KS51000	39.9				
	396	KBrH013B13			KS50960	40.3		At5	23,242,223	23,379,930
		KBrB081M20	KR50970-1	55.3	KA50970-2	43.1				
	007	KBrB060D14			KS10100	45.6		At1	1,979,125	2,081,656
	006	KBrH053G06	KS10091	58.9				At1	1,908,489	1,992,231
	004	KBrB030F10			KA10030	47.0		At1	624,039	753,147
	002	KBrB046G18	KR10010-2	78.8				At1	102,555	228,041
	179	KBrB068B07	KA30230	95.9				At3	3,332,745	3,560,299
Ax	005	KBrB013C03					1358	At1	1,510,278	1,854,223
	018	KBrS016D08					171	At1	4,444,141	4,514,309
	027	KBrH014P02					169	At1	6,154,635	6,284,133
	053	KBrB080J22					368	At1	11,470,259	11,560,904
	054	KBrB019O20					1313	At1	11,655,771	11,773,532
	057	KBrH004M10					677	At1	17,569,144	17,729,692
	059	KBrB049A09					1323	At1	18,039,565	18,156,607
	074	KBrH092K14					singleton	At1	23,559,081	23,653,002

086	KBrB064F02	singleton	At1	26,259,002	26,352,150
107	KBrB008C11	275	At2	429,541	552,369
113	KBrB057N22		At2	6,913,625	7,129,297
124	KBrH001N23		At2	10,075,679	10,112,467
125	KBrB038O14	178	At2	10,483,714	10,691,816
138	KBrB047H21	67	At2	14,223,930	14,525,726
187	KBrB021B05	1321	At3	5,267,088	5,342,493
199	KBrB004O19	447	At3	7,050,673	7,139,490
214	KBrB090B08	singleton	At3	9,702,439	9,801,277
239	KBrB088E10	singleton	At3	21,047,155	21,162,252
259	KBrB002P01	92	At4	1,332,080	1,525,404
266	KBrB019J08	445	At4	7,260,892	7,350,059
267	KBrB043N17	425	At4	8,003,112	8,050,006
277	KBrB080C12		At4	9,888,280	10,002,373
281	KBrB065H17	387	At4	10,455,646	10,590,484
306	KBrH001F03	singleton	At4	14,632,330	14,726,328
384	KBrB024J16	193	At5	19,738,736	19,892,891
406	KBrB054D07	128	At5	24,870,297	24,938,053

[a]For genetic mapping, two mapping populations, JWF3P (Kim et al., Genetics 2006, 174: 29-39) and VCS (http://www.brassica-rapa.org, unpublished our data), were used. Data matrix was frozen as 2008_v2 map.

[b]Physical map Build 2.1 (Mun et al., BMC Genomics 2008, 9: 280) was used for physical contig information.

[c]Putative counterpart regions on the *A. thaliana* genome was identified by *in silico* allocation of BAC sequence contigs by BLASTZ match of BES at a cutoff of $<E^{-6}$.

FISH, fluorescent *in situ* hybridization; Chr., chromosome; cM, centi Morgan.

Table S3. Statistics of microsynteny in the synteny blocks identified by a genome comparison of *B. rapa* and *A. thaliana* .

Number	Median E value	Number of gene[a]			Genomic events of *B. rapa*[b]			
		B. rapa	*A. thaliana*	conserved	Conservation	Loss	Translocation	Gain
227	$1E^{-151}$	10,458	16,185	6,555	52%±13%	33%±13%	5%±4%	10%±6%

[a] Tandem duplicated genes were considered as a single homolog.

[b] Values are average ± standard deviation.

Table S4. Identification of sister blocks produced by the same polyploidy events in the *Br* genome based on *At-At* and *At-Br* relationships. Degree of gene conservation is defined as described in Materials and Methods where tandem duplicated genes are considered as a single homolog.

At duplication[a] block ID	At1		At2		Cordinate file	Event[b]	Br1			Br2			Overall gene no.			non-redundant gene no.c			Degree of conservation[d]
	Start	End	Start	End			Contig	Start ID	End ID	Contig	Start ID	End ID	Br1	Br2	conserved	Br1	Br2	conserved	
A01N001a	AT1G02380	AT1G03080	AT4G01960	AT4G02710	du-01-02	3R	Br003	98	119	Br258	2	15	22	14	6	19	13	4	25.0%
					du-01-03	3R	Br004	4	25	Br258	2	15	21	14	11	18	13	5	32.3%
A02N001a	AT1G06640	AT1G07140	AT2G30840	AT2G30060	du-02-01	3R	Br006	2	36	Br134	88	103	30	16	4	28	16	3	13.6%
					du-02-02	3R	Br009	1	32	Br134	16	134	32	115	6	32	115	6	8.2%
A04N001a	AT1G13960	AT1G14420	AT2G03340	AT2G02720	du-04-01	3R	Br019	24	50	Br109	3	21	27	18	24	20	12	6	37.5%
A05N001a	AT1G15500	AT1G27130	AT1G80850	AT1G69320	du-05-01	3R	Br024	15	29	Br105	4	49	15	42	6	13	42	5	18.2%
					du-05-04	3R	Br028	23	32	Br096	17	32	10	16	4	10	16	4	30.8%
					du-05-05	3R	Br030	27	92	Br100	6	45	50	36	7	49	35	6	14.3%
					du-05-06	3R	Br031	3	30	Br101	7	27	26	20	6	25	19	5	22.7%
					du-05-07	3R	Br032	4	75	Br101	30	58	61	25	14	60	24	13	31.0%
					du-05-08	3R	Br034	2	39	Br102	13	69	31	52	11	26	51	8	20.8%
					du-05-09	3R	Br036	49	60	Br090	12	30	10	17	8	6	15	5	47.6%
					du-05-09	3R	Br036	3	14	Br091	2	31	11	29	5	9	27	3	16.7%
					du-05-10	3R	Br037	13	38	Br088	1	18	23	17	9	22	15	6	32.4%
					du-05-11	3R	Br039	14	76	Br085	26	62	53	36	11	51	36	10	23.0%
A08N001a	AT1G50090	AT1G53110	AT3G20350	AT3G15340	du-08-01	3R	Br061	1	21	Br198	17	36	21	18	5	19	18	4	21.6%
					du-08-01	3R	Br062	18	30	Br198	8	14	12	6	4	11	5	3	37.5%
					du-08-02	3R	Br064	1	53	Br186	1	26	51	25	11	50	23	6	16.4%
A10N001a	AT2G18010	AT2G25600	AT4G39490	AT4G28480	du-10-02	3R	Br117	5	40	Br318	12	54	32	41	6	32	41	6	16.4%
					du-10-03	3R	Br126	21	45	Br312	5	65	25	58	9	24	57	8	19.8%
					du-10-04	3R	Br121	2	19	Br303	1	31	16	28	5	15	27	4	19.0%
A11N001a	AT2G40820	AT2G43500	AT3G56480	AT3G59580	du-11-01	3R	Br155	3	31	Br240	11	43	29	30	6	29	30	6	20.3%
					du-11-02	3R	Br156	4	40	Br242	3	37	74	32	8	70	32	7	13.7%
					du-11-02	3R	Br156	38	74	Br243	5	38	68	34	8	66	33	7	14.1%
					du-11-02	3R	Br158	4	38	Br245	11	53	35	42	7	32	41	4	11.0%
A12N001a	AT2G01220	AT3G05960	AT5G15090	AT5G27850	du-12-01	3R	Br169	2	23	Br347	16	68	42	50	8	42	50	8	17.4%
A14N001a	AT3G09500	AT3G11910	AT5G02610	AT5G06600	du-14-01	3R	Br176	22	34	Br328	6	32	12	27	5	12	27	5	25.6%
					du-14-03	3R	Br178	7	53	Br330	7	32	44	24	7	44	24	7	20.6%
					du-14-04	3R	Br180	1	35	Br332	50	83	32	31	9	31	30	8	26.2%
					du-14-05	3R	Br181	16	41	Br332	31	57	20	27	5	20	27	5	21.3%
A21N001a	AT4G17460	AT4G19050	AT5G47370	AT5G45510	du-21-01	3R	Br275	3	32	Br381	6	25	30	20	8	30	20	8	32.0%
					du-21-04	3R	Br280	6	61	Br379	37	83	54	46	8	53	45	7	14.3%
A22N001a	AT4G24630	AT4G27800	AT5G56860	AT5G53140	du-22-03	3R	Br299	10	40	Br393	8	54	28	43	6	27	43	5	14.3%
A01N001a	AT1G02890	AT1G03120			tr-01-01	4R	Br003	96	126	Br004	2	34	31	32	23	24	25	12	49.0%
A05N001a	AT1G26790	AT1G27110			tr-05-01	4R	Br039	62	82	Br040	18	21	17	4	4	17	4	4	38.1%
					tr-05-01	4R	Br041	5	37	Br040	3	10	30	8	9	26	8	7	41.2%
A10N001a	AT4G36640	AT4G36140			tr-10-01	4R	Br319	13	37	Br318	7	47	22	39	12	18	37	9	32.7%

	AT4G33580	AT4G33250			tr-10-02	4R	Br313	8	23	Br314	113	139	16	24	10	16	24	10	50.0%
A11N001a	AT3G59200	AT3G59300			tr-11-01	4R	Br245	49	60	Br244	16	24	10	9	8	7	5	3	50.0%
	AT3G59650	AT3G59830			tr-11-01	4R	Br245	7	26	Br246	26	38	19	13	8	17	13	7	46.7%
	AT3G61570	AT3G61840			tr-11-01	4R	Br249	2	46	Br250	8	32	40	24	12	40	24	12	37.5%
A13N001a	AT3G07540	AT3G07630			tr-13-01	4R	Br174	2	14	Br175	72	89	13	18	10	13	18	10	64.5%
A14N001a	AT3G11540	AT3G11630			tr-14-01	4R	Br180	23	33	Br181	33	43	10	9	8	10	9	8	84.2%
	AT5G05780	AT5G05980			tr-14-02	4R	Br331	20	50	Br332	78	89	31	12	13	23	12	7	40.0%
A16N001a	AT3G27690	AT3G28040			tr-16-01	4R	Br217	16	23	Br218	4	32	8	29	10	6	27	6	36.4%
	AT3G28180	AT3G28500			tr-16-02	4R	Br217	1	13	Br219	2	32	12	28	20	8	21	6	41.4%
A18N001a	AT5G60330	AT5G60120			tr-18-01	4R	Br401	2	35	Br402	3	22	32	19	26	26	17	6	27.9%
A23N001a	AT5G09920	AT5G10420			tr-23-01	4R	Br338	25	50	Br339	2	32	25	29	19	22	27	14	57.1%
A24N001a	AT5G11810	AT5G11880			tr-24-01	4R	Br341	22	39	Br342	34	44	15	11	7	13	11	6	50.0%
A26N001a	A5G60330	AT5G60120			tr-26-01	4R	Br401	2	35	Br402	3	22	32	19	26	26	17	6	27.9%
					tr-xx-02	4R	Br370	3	25	Br371	1	19	23	17	9	23	17	9	45.0%
					se-xx-02	S	Br355	1	14	Br354	3	17	13	15	18	12	12	10	83.3%

[a]Information for the duplicated blocks in the *At* genome was based on the report of Bowers et al. (Nature 2003, 422: 433-438).

[b]3R, the most recent duplication event; 4R, triplication event; S, segmental duplication event.

[c]Tandem duplicated genes are considered as a single homolog.

[d]Degree of conservation is calculated by division of conserved gene number by total gene number.

Table S5. Identification of sister blocks produced by the same polyploidy events in the *Bo* genome. Degree of gene conservation is defined as described in Materials and Methods where tandem duplicated genes are considered as a single homolog.

At duplication[a] block ID	At1		At2		Event[b]	Bo1			Bo2			Overall gene no.			non-redundant gene no.c			Degree of conservation[d]
	Start	End	Start	End		Contig	Start ID	End ID	Contig	Start ID	End ID	Bo1	Bo2	conserved	Bo1	Bo2	conserved	
A21N001a	AT4G17160	AT4G17880	AT5G47770	AT5G46760	3R	A	A.m00005	A.m00087	D	D.m00003	D.m00027	35	24	9	35	24	9	30.5%
					3R	A	A.m00595	A.m00086	E	E.m00604	E.m00067	14	11	5	14	11	5	40.0%
					3R	A	A.m00005	A.m00038	F	F.m00002	F.m00071	22	20	5	22	20	5	23.8%
					3R	B	B.m00407	B.m00428	D	D.m00003	D.m00069	27	23	10	24	23	9	38.3%
					3R	B	B.m00415	B.m00428	E	E.m00589	E.m00067	14	10	8	12	10	7	63.6%
					3R	B	B.m00407	B.m00414	F	F.m00010	F.m00071	15	16	7	13	16	6	41.4%
					3R	C	C.m00002	C.m00062	D	D.m00003	D.m00020	24	15	7	23	15	6	31.6%
					3R	C	C.m00431	C.m00062	E	E.m00604	E.m00030	7	4	3	7	4	3	54.5%
					3R	C	C.m00002	C.m00448	F	F.m00009	F.m00071	21	17	8	20	17	7	37.8%
A21N001a	AT5G47770	AT5G46760			4R	A	A.m00016	A.m00086	B	B.m00407	B.m00428	27	27	18	27	24	17	66.7%
					4R	A	A.m00016	A.m00057	C	C.m00002	C.m00062	16	24	15	16	23	14	71.8%
					4R	B	B.m00407	B.m00042	C	C.m00002	C.m00062	18	24	15	15	23	11	57.9%
A21N001a	AT4G17160	AT4G17880			4R	D	D.m00019	D.m00030	E	E.m00589	E.m00594	13	12	5	13	12	5	40.0%
					4R	D	D.m00003	D.m00019	F	F.m00002	F.m00071	14	20	10	14	20	10	58.8%
					4R	D	D.m00030	D.m00062	G	G.m00005	G.m00047	17	16	13	16	14	11	73.3%

[a]Information for the duplicated blocks was based on the report of Town et al. (Plant Cell 2006, 18: 1348-1359).

[b]3R, the most recent duplication event; 4R, triplication event.

[c]Tandem duplicated genes are considered as a single homolog.

[d]Degree of conservation is calculated by division of conserved gene number by total gene number.

Table S6. Sources of genomic and transcript sequences used in this study.

Species	Data source	Download URL	Sequence type
Arabidopsis thaliana	NCBI NR database	ftp://ftp.ncbi.nih.gov/genomes/Arabidopsis_thaliana/	Complete genome
Oryza sativa	TIGR Rice Database	ftp://ftp.tigr.org/pub/data/Eukaryotic_Projects/o_sativa/annotation_dbs/pseudomolecules/version_5.0/	Draft genome data, v5.0
Medicago truncatula	*Medicago truncatula* sequencing project	http://www.medicago.org/genome/downloads/Mt1/	Draft genome data, Mt1
Populus trichocarpa	DOE JGI *Populus* genome project	http://genome.jgi-psf.org/Poptr1_1/Poptr1_1.download.ftp.html	Draft genome data, v1.0
Brassica napus	NCBI dbEST database	ftp://ftp.ncbi.nih.gov/repository/dbEST/	singlepass cDNA
Brassica oleracea	NCBI dbEST database	ftp://ftp.ncbi.nih.gov/repository/dbEST/	singlepass cDNA

All data were collected in February, 2008.

NCBI, National Center for Biotechnology Information; NR, nonredundant; TIGR, The Institute of Genome Research; DOE, US Department of Energy; JGI, Joint Genome Institute; dbEST, database of expressed sequence tags.
