# Supplementary figures and images for: Genome-wide comparative analysis of the Brassica rapa gene space reveals genome shrinkage and differential loss of duplicated genes after whole genome triplication
Source: Genome Biol. 2009 Oct 12;10(10):R111. doi: 10.1186/gb-2009-10-10-r111 (PMC2784326; doi:10.1186/gb-2009-10-10-r111)

Figure S1

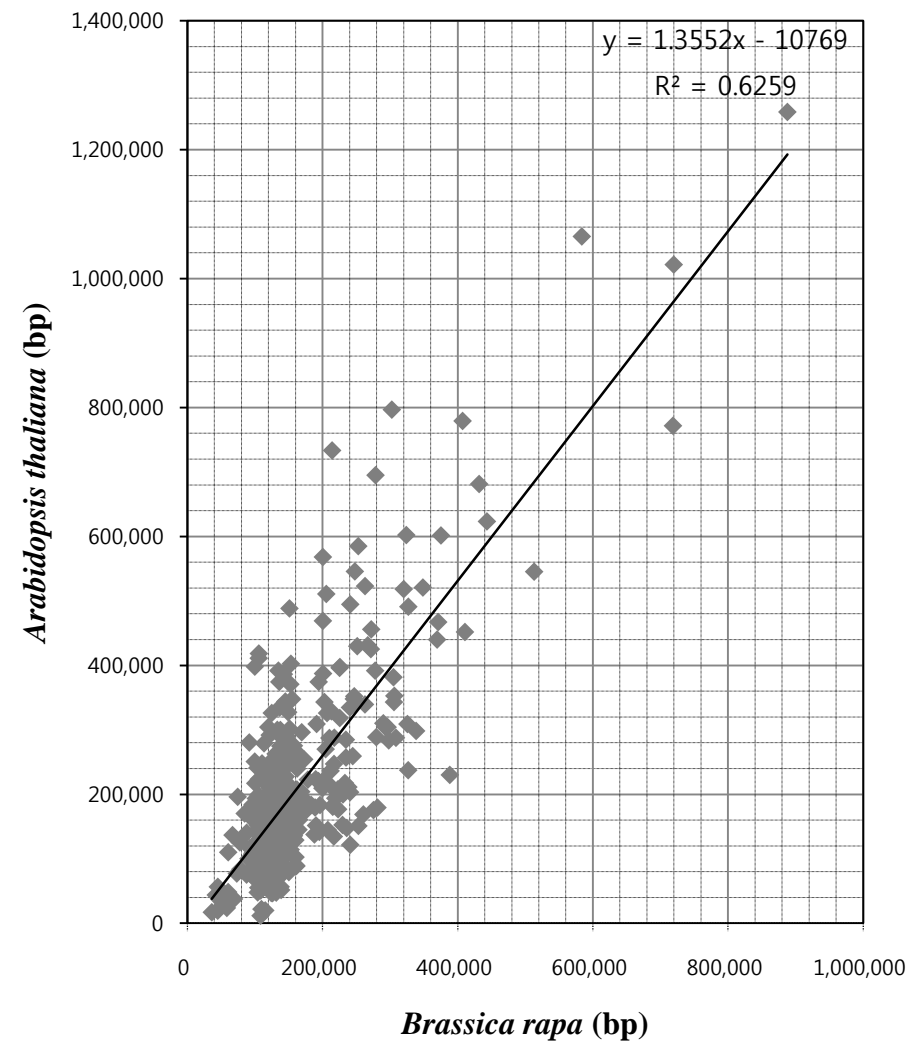

Figure S2

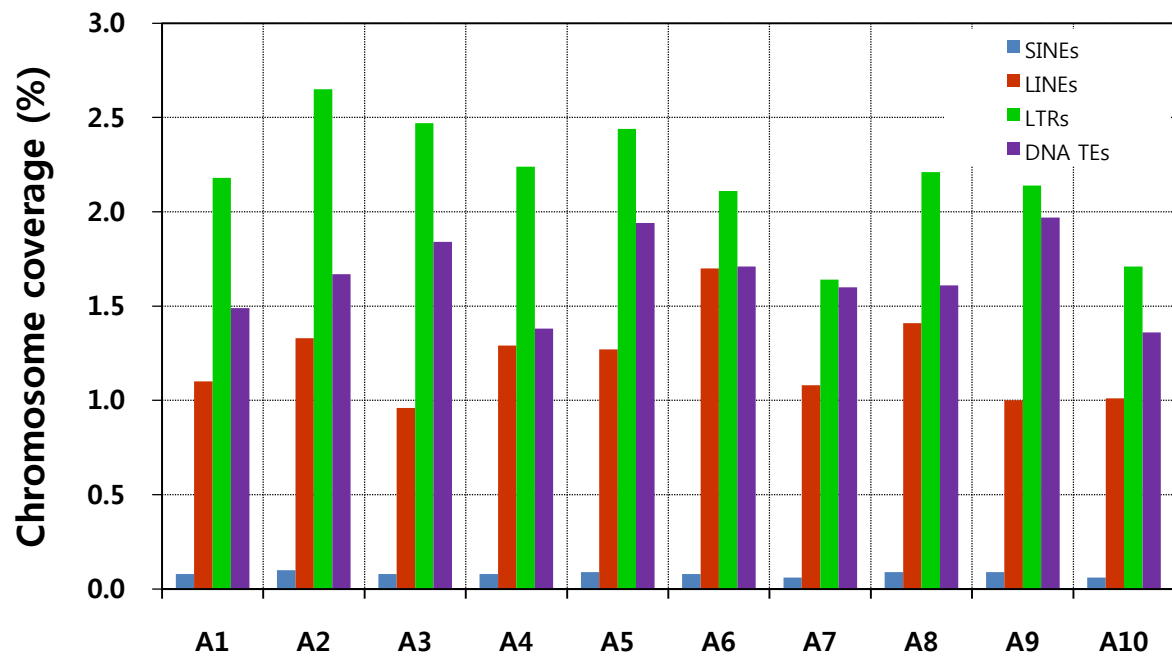

Figure S3

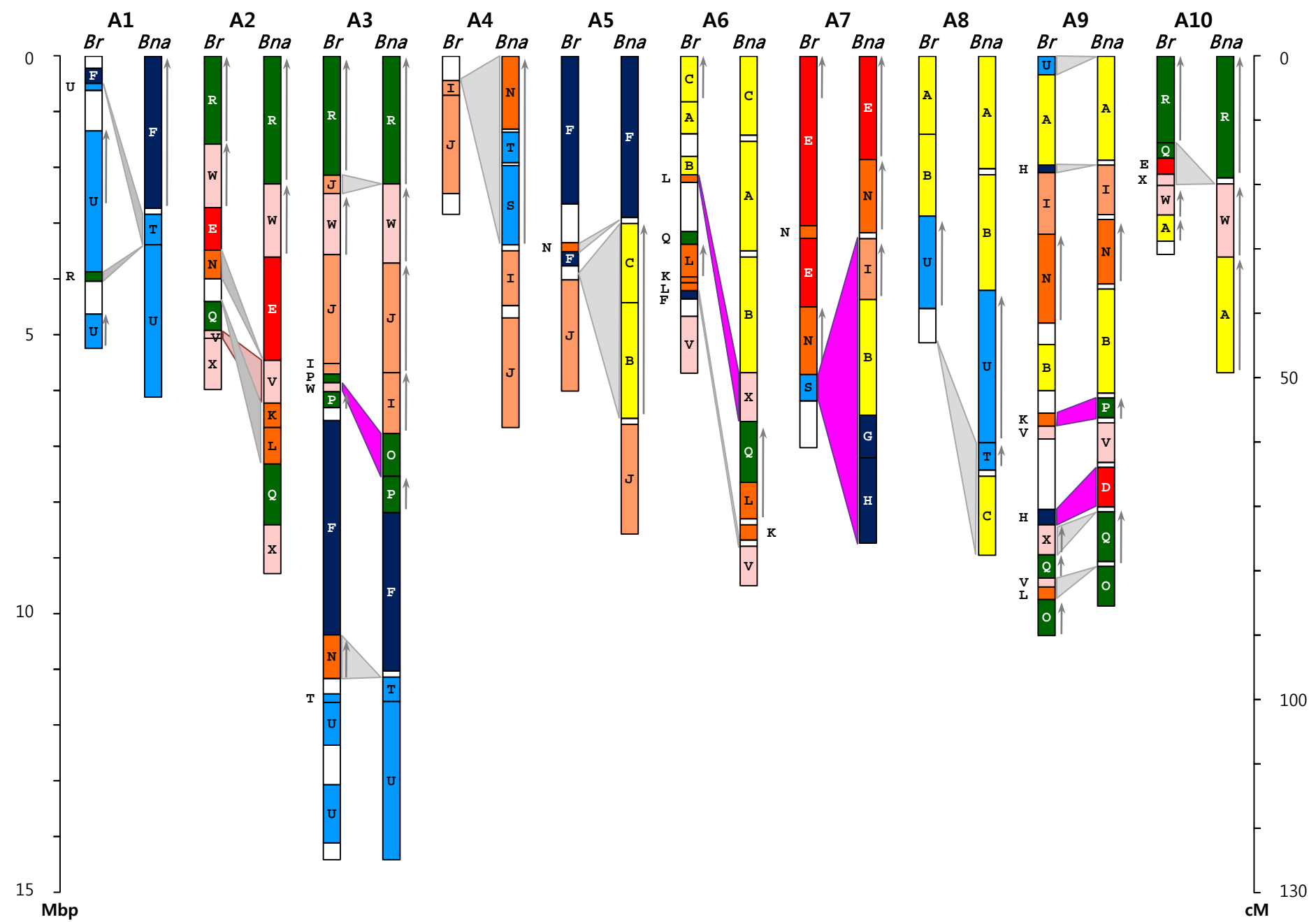

Figure S4

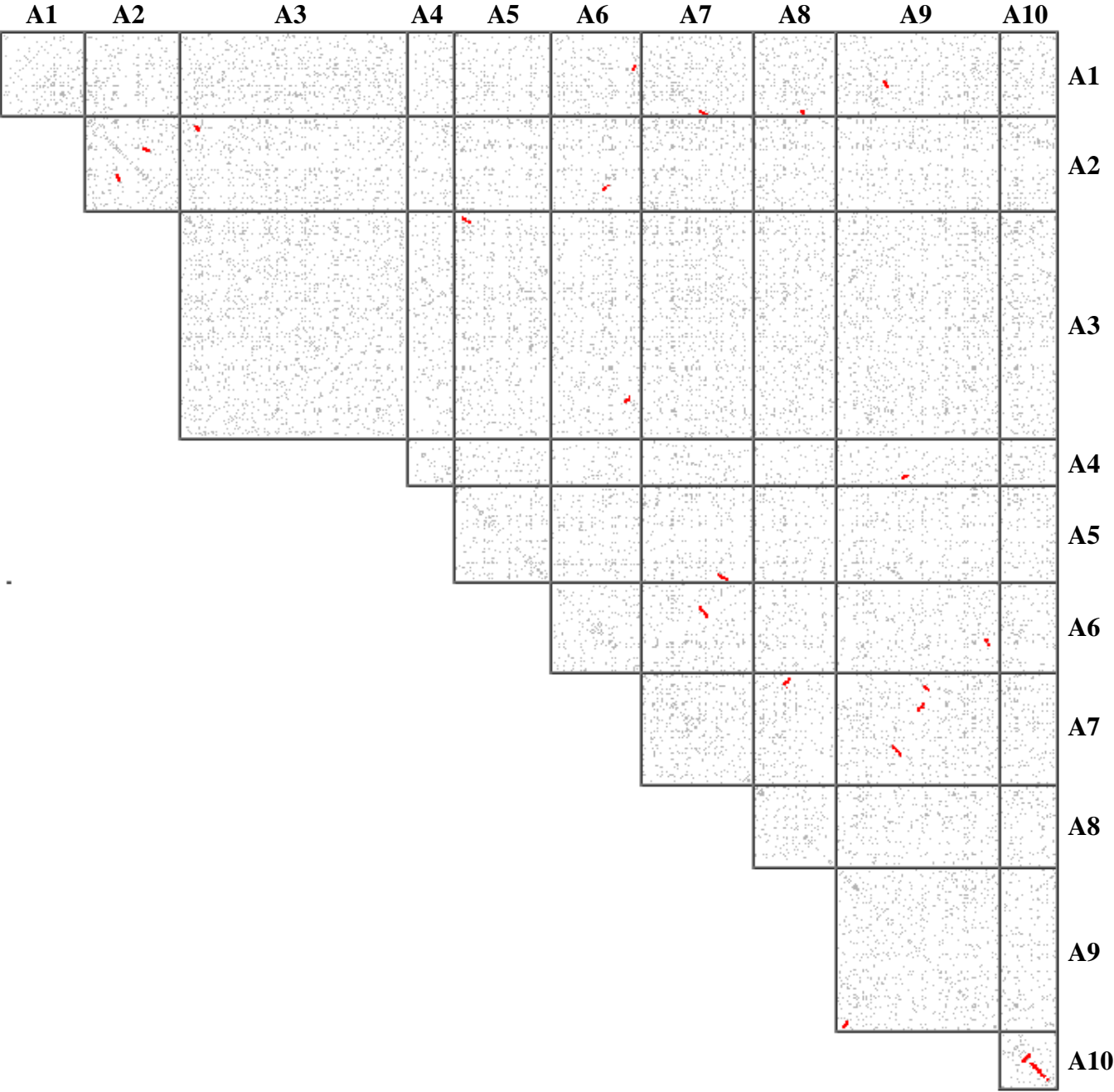

Supplement: Additional data file 2 — Figure S1: comparison of homologous block end-point distances between 410 B. rapa sequence contigs and their Arabidopsis counterpart regions, indicating a genome shrinkage of approximately 30% in B. rapa. Figure S2: abundance of different transposable element types in the B. rapa genome. Figure S3: comparison of Brassica 'A' genome structures between B. rapa and B. napus. Genome blocks were defined based on 24 AK genome building blocks. The genome structure of Bna was obtained from the reports of Parkin et al. [23] and Schranz et al. [37]. Regions characterized by significant rearrangements (pink box) or insertions/deletions (gray box) between genomes are highlighted by colored boxes. Scale bars on the margins indicate megabase-pairs (Mbp) for Br or centi-Morgans (cM) for Bna. Blocks with the opposite orientation relative to AK are indicated by a gray upward-pointing arrow on the right side of the block. Figure S4: dot plot of B. rapa compared with itself. Each dot in the dot plot represents a reciprocal best BLASTP match between gene pairs at a cutoff value of <E-20. Red dots show the regions of synteny identified by DiagHunter. [file gb-2009-10-10-r111-S2.PDF]
